# Controlling Intestinal Organoid Polarity using Synthetic Dynamic Hydrogels Decorated with Laminin‐Derived IKVAV Peptides

**DOI:** 10.1002/adhm.202502079

**Published:** 2025-09-04

**Authors:** Laura Rijns, Joost A. P. M. Wijnakker, Victor A. Veenbrink, Riccardo Bellan, Fenna W. B. Craenmehr, Simone I. S. Hendrikse, Johnick F. van Sprang, Wim de Lau, E. W. Meijer, Hans Clevers, Patricia Y. W. Dankers

**Affiliations:** ^1^ Institute for Complex Molecular Systems Eindhoven University of Technology Eindhoven 5600 MB the Netherlands; ^2^ Department of Biomedical Engineering Eindhoven University of Technology Eindhoven 5600 MB the Netherlands; ^3^ Hubrecht Institute University Medical Center (UMC) Utrecht Utrecht 3584CX the Netherlands; ^4^ Department of Chemical Engineering and Chemistry Eindhoven University of Technology Eindhoven 5600 MB the Netherlands; ^5^ The School of Chemistry and the RNA Institute University of New South Wales Sydney New South Wales 2052 Australia; ^6^ Pharma, Research and Early Development of F. Hoffmann‐La Roche Ltd 4070 Basel Switzerland

**Keywords:** dynamics, IKVAV, intestinal organoids, laminin, polarity, supramolecular hydrogel, synthetic extracellular matrices

## Abstract

Intestinal organoids are three‐dimensional cellular structures that are cultured in laminin‐rich Matrigel, yielding organoids with correct, basal‐out polarity. Removal of Matrigel results in organoids with reversed, apical‐out polarity, demonstrating its vital role. However, Matrigel's composition is ill‐defined, and its pathogenic origin poses challenges in reproducibility. Therefore, we here introduce a fully synthetic dynamic hydrogel that presents the IKVAV peptide as a laminin‐mimic for guiding intestinal organoid polarity in a minimalistic, controlled manner. The ureido‐pyrimidinone moiety is used to form supramolecular hydrogels that have orthogonal control over properties, like stiffness, ligand type and concentration. It is found that the IKVAV peptide combined with integrin activating antibody TS2/16 controls intestinal organoid polarity. Increasing hydrogel dynamics (stress‐relaxing half‐life time of ≈1000 to 30 s) further supports the growth of intestinal organoids with correct polarity, while a bulk level of stiffness (*G*’ ≈0.7 kPa) is crucial to offer mechanical support. Through manipulation of integrins, it is revealed that the IKVAV‐organoid interaction is integrin β1‐mediated. Our findings demonstrate the essential role of the IKVAV motif in guiding intestinal organoid polarity in synthetic dynamic hydrogels – paving the way for the future design of synthetic systems to culture complex living tissue.

## Graphical abstract

Design rules are presented to control intestinal organoid polarity in fully synthetic hydrogels. The laminin‐derived IKVAV sequence is crucial to obtain correct intestinal organoid polarity. Increasing hydrogel dynamics further supports the growth of correctly polarized intestinal organoids, while a bulk level of stiffness (G’ ≈ 0.7 kPa) is crucial to offer mechanical support.

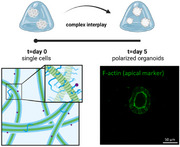

## 1 Introduction

Intestinal organoids are grown from adult stem cells and mimic key characteristics and functionalities of the human intestine.^[^ ^1^, ^2^, ^3^ ^]^ Intestinal organoids are a powerful tool for studying health and disease of the human intestine, for example for disease modeling, drug development, understanding native development and regeneration, and ultimately for personalized medicine.^[^ ^4^, ^5^, ^6^, ^7^ ^]^ Important when culturing epithelial stem cells into intestinal organoids is their apical‐basal polarity, which plays a vital role in the proper functioning of these organoids: loss of correct polarity is known to contribute to cancer progression.^[^ ^8^ ^]^ Essential basolateral proteins, like integrins, are responsible for anchoring the organoid to their extracellular surroundings, while on the apical side,^[^ ^9^ ^]^ proteins like ezrin connect the cell membrane to the actin cytoskeleton.^[^ ^10^ ^]^

Intestinal stem cells cultured in Matrigel – a basement membrane matrix secreted by Engelbreth–Holm–Swarm (EHS) mouse tumors^[^ ^11^, ^12^, ^13^ ^]^ – grow into organoids with the correct basal‐out polarity.^[^ ^2^, ^14^, ^15^, ^16^, ^17^ ^]^ Removal of the supportive matrix and culture in suspension yields organoids with reversed, apical‐out polarity, demonstrating the crucial role of the surrounding matrix in directing organoid polarity.^[^ ^18^, ^19^ ^]^ Zooming in on Matrigel, it mainly constitutes of laminin‐111 (≈60%) and together with collagen‐IV, nidogen and perlecan, they occupy more than 90% of the total protein content of Matrigel. Especially laminin‐111 and collagen‐IV are important, as both extracellular matrix (ECM) proteins are components of the in‐vivo basement membrane and capable of inducing and maintaining correct apical‐basal epithelial polarity.^[^ ^20^, ^21^ ^]^ However, the number and concentration of those individual components in Matrigel are not exactly known, which poses challenges in isolating the factors that drive intestinal organoid polarity.

In the urge to find a replacement for Matrigel to grow intestinal organoids with the correct apical‐basal polarity in a controlled manner, several hydrogels have been developed, ranging from completely natural to hybrid to fully synthetic versions, each with their own advantages and limitations.^[^ ^22^, ^23^, ^24^, ^25^, ^26^, ^27^, ^28^, ^29^ ^]^ Among the many alternatives resembling Matrigel, natural‐based hydrogels are often used, where collagen was successfully used to afford correctly polarized organoids through targeting integrin α2β1, an abundantly expressed integrin receptor on intestinal stem cells.^[^ ^30^, ^31^, ^32^, ^33^ ^]^ The Garnier, Schwanck and Spence labs successfully grew intestinal organoids both in non‐adhesive alginate hydrogels as well as in bioactive RGD‐functionalized nanocellulose or fibrin‐based hydrogels. Interestingly, the Chaudhuri lab used alginate gels to fundamentally understand apical‐basal formation in a human epiblast model.^[^ ^34^ ^]^ They reveal that apical actin polymerization generates a force which is the origin for lumen expansion in epiblasts – crucial information which can and should be used to understand and control intestinal organoid polarity in designer hydrogels. Thermo‐responsive collagen‐nanocellulose hydrogels were also developed, aiming to facilitate organoid splitting based on temperature‐controlled gel–sol transitions of the matrix.^[^ ^35^, ^36^, ^37^, ^38^ ^]^ In addition, the Heilshorn lab successfully grew intestinal organoids in their hydrogels based on elastin‐like proteins (ELPs), and showed that organoid growth was optimal in gels with a stiffness of (*G*’ ≈ 1 kPa), a slow stress relaxation rate (τ_1/2_ ≈18 h), and medium to high integrin ligand concentration (0.5–1 mm RGD adhesive peptide).^[^ ^39^ ^]^ Overall, such natural hydrogels generally exhibit excellent bioactivity but lack control over various hydrogel properties.^[^ ^40^, ^41^, ^42^ ^]^ Consequently, synthetic alternatives are being developed as substitutes for these poorly defined matrices.^[^ ^25^ ^]^

Synthetic hydrogels, particularly those based on poly(ethylene glycol) (PEG), were successfully mixed with natural bioactive polymers like fibronectin, laminin, collagen, hyaluronic acid or perlecan, yielding hybrid matrices, which present excellent bioactivity combined with control over mechanical properties.^[^ ^43^ ^]^ Fully synthetic hydrogels have been covalently functionalized with short sequences, like RGD, PHSRN (fibronectin mimicking) or GFOGER (collagen mimicking), to introduce bioactivity, as successfully demonstrated by the Lutolf, Garcia, Griffith, and our own laboratory.^[^ ^40^, ^44^, ^45^, ^46^, ^47^, ^48^, ^49^, ^50^ ^]^ Other synthetic polymers were also successfully used to grow intestinal organoids, such as poly (lactic‐*co*‐glycolic acid) by the Wang lab.^[^ ^51^ ^]^ However, all of these covalent matrices lack (control over) dynamics. Recently, the Lutolf lab introduced stress relaxation behavior into synthetic hydrogels through cytosine hydrogen bonding.^[^ ^52^ ^]^ Their supramolecular, stress‐relaxing hydrogels outperformed the covalent hydrogels because of their ability to dissipate tissue forces, allowing the growth of single cells into multicellular intestinal organoids, and facilitating morphogenesis.

Besides the importance of hydrogel dynamics, the presented bioactive cue impacts intestinal organoid behavior.^[^ ^24^, ^37^, ^43^, ^53^ ^]^ As discussed above, epithelial cells possess a correct basal‐out polarity when cultured in laminin^[^ ^20^ ^]^ and this polarity is therefore hypothesized to be crucial in guiding intestinal organoid polarity. When comparing epithelial cells in intestinal organoids to other cell types, like neuronal cells, they also need an ECM to guide neuronal development, migration, and synapse formation.^[^ ^54^ ^]^ For neurons, laminin has also been revealed crucial to promote neural outgrowth, survival, and network formation through interacting with cell surface receptors, like integrins.^[^ ^55^ ^]^ Recently, the minimal synthetic peptide sequence IKVAV was successfully used as a laminin‐mimic to support neural growth, maturation, differentiation, and regeneration, as demonstrated by the Stupp laboratory.^[^ ^56^, ^57^, ^58^, ^59^ ^]^ Based on these results, the IKVAV sequence as synthetic mimic of laminin is hypothesized to be a potent candidate to guide intestinal organoid behavior in synthetic matrices.

Supramolecular hydrogels based on non‐covalent interactions have great potential as ECM mimics to grow intestinal organoids in fully synthetic matrices, due to their intrinsic dynamics and the ability to orthogonally control hydrogel properties. In our laboratories, we have ample expertise using the ureido‐pyrimidinone (UPy) moiety as a supramolecular monomer^[^ ^60^, ^61^ ^]^ to formulate supramolecular polymeric hydrogels. These supramolecular UPy‐based hydrogels are used as ECM mimics to grow cells, spheroids, and organoids.^[^ ^40^, ^41^, ^42^, ^50^ ^]^ At the nanometer level, the UPy moiety forms quadruple hydrogen bonding to form dimers. When equipped with additional urea groups, aliphatic linkers, and an oligo(ethylene glycol) (OEG) linker, fiber formation in the lateral direction is facilitated in aqueous environments (monofunctional UPy, **M‐UPy**). Bioactivity is introduced through the covalent attachment of minimal synthetic sequences to **M‐UPy**, such as cyclic RGD (cRGD) (yielding **UPy‐cRGD**) as a fibronectin mimic^[^ ^40^, ^41^, ^50^ ^]^ or GFOGER (yielding **UPy‐GFOGER**) as a collagen mimic.^[^ ^49^, ^62^ ^]^ The bioactive monomers are mixed with unfunctionalized **M‐UPy** monomers to create bioactive co‐assembled supramolecular fibers. To transition from fibers to the hydrogel state, a highly dynamic bifunctional UPy (**B‐UPy**) is incorporated as a supramolecular cross‐linker to cross‐link the monofunctional fibers non‐covalently, initiating the formation of a 3D transient hydrogel network.^[^ ^42^, ^63^, ^64^ ^]^

Importantly, as these supramolecular UPy hydrogels are intrinsically dynamic, they offer degradation‐independent dynamic behavior. This dynamic behavior is hypothesized to support the growth of single stem cells into multicellular intestinal organoids, through dissipating tissue forces exerted on the synthetic matrix. These dynamics arise on different length and time scales: the molecular dynamics occur at the molecular level with monomer exchange within supramolecular fibers (length scale ≈nm), while bulk dynamics (stress relaxation) are observed at the macroscopic level with fibers rearranging in the hydrogel (length scale ≈µm to mm).^[^ ^41^, ^65^ ^]^ We have previously shown that the molecular dynamics (monomer exchange) is rather slow in UPy supramolecular fibers, with only 10% of monomer exchange in a 1 h timeframe, ensuring the robust incorporation of the bioactive supramolecular monomers within the supramolecular fibers.^[^ ^40^, ^41^ ^]^ While stress relaxation (τ_1/2_ ≈s to min) on the macroscopic level enables the dynamic recruitment of bioactive fibers, leveraging multivalent effects and is therefore hypothesized to greatly support the growth of single stem cells into multicellular organoids through the dissipation of tissue forces.

**Figure 1:**
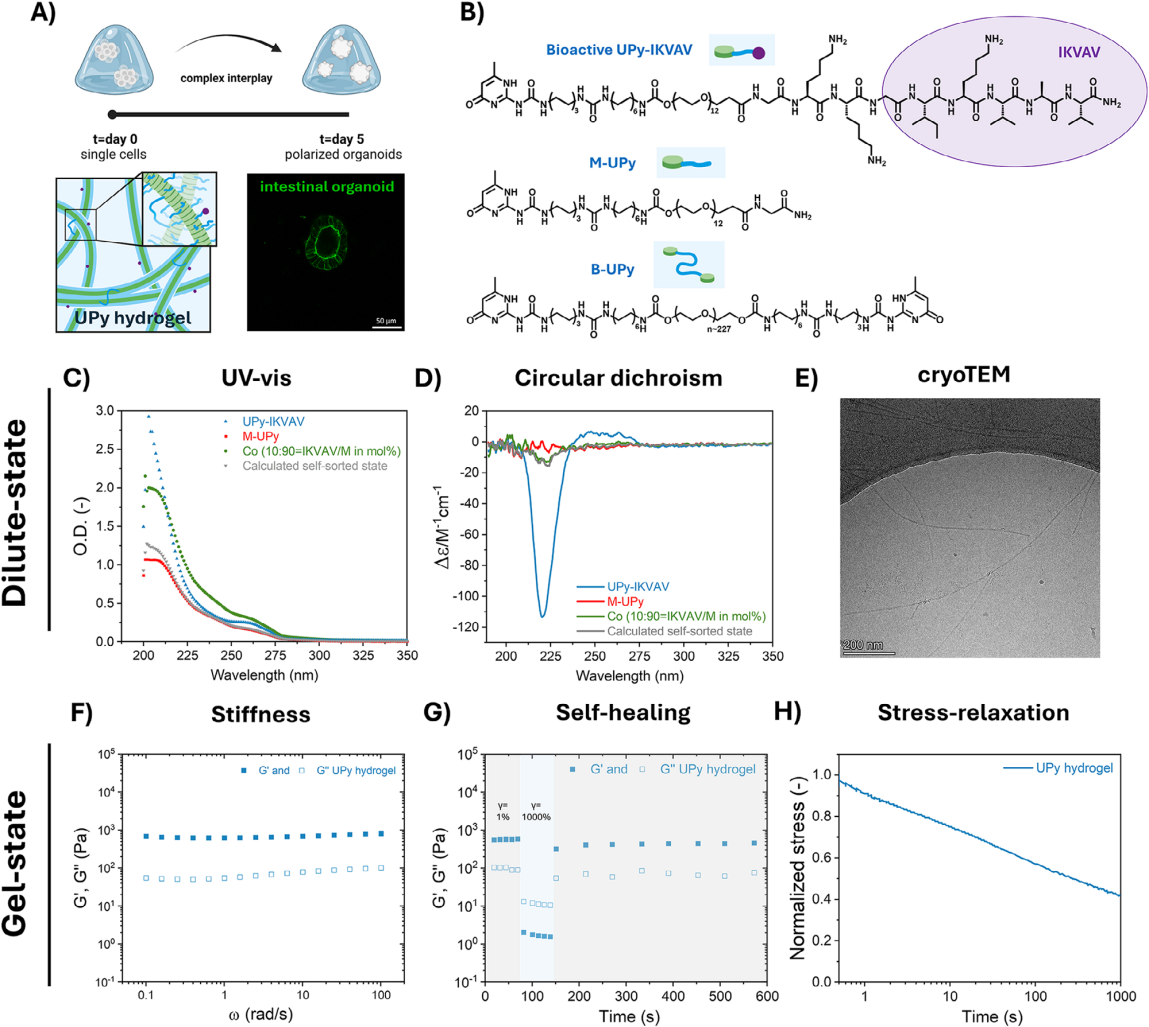
Aim of the study to control intestinal organoid polarity using an IKVAV‐presenting synthetic, dynamic hydrogel, including the molecular design of the supramolecular hydrogel system and gel‐and dilute‐state characterization. A) Schematic overview of our aim: single stem cells are grown into human intestinal organoids with correct polarity in the presence of the laminin‐minimalistic sequence IKVAV. Scale bar = 50 µm. B) Molecular structures of the supramolecular monomers within the supramolecular hydrogel network: non‐functional **M‐UPy** and bioactive UPy ligands are copolymerized to form monofunctional, bioactive fibers. Upon including **B‐UPy** that has two UPy groups on its periphery, the monofunctional **M‐UPy** fibers are cross‐linked to form a transient hydrogel network. The resulting synthetic supramolecular hydrogels have orthogonal tunable mechanical, dynamical and bioactive properties. C–E) Characterization in dilute‐state (c ≈50–250 µm range), using a similar mixing ratio between **M UPy** and **UPy‐IKVAV** for the co‐assemblies as used in the gel‐state (**M‐UPy**/**UPy‐IKVAV** = 90/10 mol %), with C) ultraviolet‐visible (UV–vis) spectroscopy measurements of pure **M‐UPy**, pure **UPy‐IKVAV** and the co‐assembly (both experimentally measured and the expected co‐assembled state), D) circular dichroism (CD) measurements of pure **M‐UPy**, pure **UPy‐IKVAV** and the co‐assembly (both experimentally measured and the expected co‐assembled state), E) cryo transmission electron micr image of co‐assembly (c_total_ = 250 µm). F–H) Characterization of supramolecular hydrogels in gel‐state at c = 1.2 w/v% with 1 mm **UPy‐IKVAV**, with a molar ratio between **M‐UPy**/**UPy‐IKVAV** = 90/10 mol % for the co‐assemblies. F) Frequency‐dependent stiffness (γ = 1%), G) self‐healing behavior (with γ = 1% and ω = 1 rad s^−1^ in “relaxed state,” and γ = 1000% and ω = 1 rad s^−1^ in ‘stressed state‘ and H) stress relaxation behavior (γ = 7.5%).

Both epithelial and neuronal cells rely on laminin in the ECM for healthy development and survival.^[^ ^20^, ^21^, ^54^, ^55^ ^]^ Inspired by the successful use of the laminin‐derived IKVAV peptide to support neuronal growth, survival and regeneration,^[^ ^56^, ^57^ ^]^ we here report on intrinsically dynamic, supramolecular hydrogels that present the laminin‐derived IKVAV peptide to guide intestinal organoid polarity in a minimalistic and fully controlled fashion (**Figure** **1**A). The supramolecular nature of our hydrogel system allows to orthogonally tune ligand type, ligand concentration, dynamics as well as stiffness. Through supramolecular tuning of these parameters, we present design rules for synthetic matrices to control the intestinal organoid‐hydrogel interaction. Additionally, we investigate the origin of the IKVAV‐organoid interaction through manipulating integrins. Future work may utilize these guidelines to culture complex living tissue in a controlled fashion, useful for future personalized drug screening applications.

## 2 Results and Discussion

### 2.1 Molecular Design and Synthesis of Supramolecular Monomers

The modular nature of our supramolecular approach allows for orthogonal control over hydrogel bioactivity, stiffness and dynamics. Regarding hydrogel bioactivity, the laminin‐derived IKVAV peptide was covalently attached to **M‐UPy**, with synthetic details reported in the (Schemes S1 and S2, Supporting Information), yielding pure **UPy‐IKVAV** as a fluffy white solid with a yield of ≈51% as confirmed with liquid chromatography followed by mass spectrometry (LC‐MS) (Figures S1 and S2, Supporting Information). A GKKG sequence was installed between **M‐UPy** and IKVAV to ensure water‐solubility. The synthesis of **M‐UPy** and **B‐UPy** are performed as previously reported.^[^ ^50^ ^]^

### 2.2 Formulation of IKVAV‐Containing Supramolecular Fibers and Hydrogels

#### 2.2.1 Supramolecular Formulation of IKVAV‐Containing Supramolecular Fibers in Dilute‐State

An adequate amount of solid **M‐UPy** powder was first weighed into a clean glass vial, then dissolved in 80 mm NaOH in phosphate‐buffered saline (PBS) and heated to 70 °C for 30 min while stirring. During this process, part of the hydroxy groups of the UPy enol tautomer are deprotonated, yielding less associated molecules. After cooling the solution to room‐temperature, it was neutralized with 1 m HCl in PBS to reach physiological pH. **UPy‐IKVAV** was dissolved in PBS and heated to 70 °C for 15 min. **M‐UPy** and **UPy‐IKVAV** were combined under neutral conditions to achieve the desired concentrations. Stock solutions were prepared at a total concentration of 1 mm and stored at 4 °C until further use. Spectroscopic measurements were performed at a total UPy concentration of 50 µm and cryo transmission electronic microscopy (cryoTEM) measurements at 250 µm, with a similar molar ratio between **M‐UPy**/**UPy‐IKVAV** as used in the regular supramolecular hydrogels, which is 90/10 (mol %) for **M‐UPy**/**UPy‐IKVAV**. The 1 mm stock solutions were diluted using PBS with pH 7.4 to obtain the 50 or 250 µm target concentrations. Samples were stored at 4 °C for two nights before performing UV and CD analysis measurements.

#### 2.2.2 Supramolecular Formulation of IKVAV‐Containing Supramolecular Hydrogels in Gel‐State Including Cell Encapsulation Procedure

To formulate the supramolecular UPy hydrogels^[^ ^66^ ^]^ and for encapsulation of the stem cells inside the hydrogels to allow the growth of organoids in 3D, a strict formulation procedure was used. In short, **M‐UPy** was dissolved under basic conditions and elevated temperature (70 °C) for 15 min at the desired stock concentration. **UPy‐IKVAV** was dissolved in neutral PBS at 70 °C for 15 min. Then, bioactive **UPy‐IKVAV** was combined with **M‐UPy** solution under basic conditions to ensure homogeneous mixing. The combined bioactive **M‐UPy** solution was then neutralized. **B‐UPy** was dissolved in neutral PBS at elevated temperature (70 °C) at its desired stock concentration. The organoids, pre‐cultured in basement membrane extract (BME) – an alternative to Matrigel – were dissociated toward single cells using a mixed mechanical and enzymatic approach (with details in Experimental Section). Stem cells were then encapsulated inside **M‐UPy** solution in a 2:3 volume ratio (i.e., 20 µL cells with 30 µL **M‐UPy** solution). **B‐UPy** solution was then pipetted into a U‐shaped 96‐well plate (to facilitate scooping out of the hydrogel from the wells for later characterization) and mixed with the **M‐UPy** solution containing the cells in a 1:5 volume ratio (i.e., 10 µL **B‐UPy** with 50 µL **M‐UPy** solution containing the cells). Stem cells encapsulated inside the UPy hydrogels were left for equilibration in the incubator at 37 °C for ≈1–2 h (depending on the hydrogel concentration), after which culture medium was added in a very careful, dropwise manner. During the first 2 days of culture, Rho‐kinase (ROCK) inhibitor was added in both the cell suspension and the supplemented medium. Likewise, other pharmacological treatments that were carried out in this study (integrin activating antibody TS2/16 and integrin inhibiting antibody AIIB2) were added in both the cell suspension and the supplemented medium. Both integrin manipulators were refreshed every 2 days during the course of the experiment.

Importantly, as the separate supramolecular precursors are all solutions, this allows for the encapsulation of the stem cells in 3D upon rapid formation of a supramolecular hydrogel under physiological conditions. For the mechanical and dynamical characterization of the UPy hydrogels, hydrogels were equilibrated overnight at 4 °C and sealed with parafilm to prevent evaporation, after which rheological experiments were performed the next day.

Control over hydrogel stiffness is realized by varying the total UPy hydrogel concentration, while keeping a fixed ratio between the supramolecular monomers as well as a fixed absolute ligand concentration of 1 mm **UPy‐IKVAV**. Control over hydrogel dynamics is achieved by tuning the ratio between the monofunctional (M) and bifunctional (B) supramolecular monomers, while also keeping a fixed absolute ligand concentration of 1 mm **UPy‐IKVAV**. The molecular compositions and corresponding concentrations used throughout the different experiments are depicted in Table S1–S5 (Supporting Information).

### 2.3 Characterization of UPy‐IKVAV‐Containing Supramolecular Solution and Hydrogels

We first characterized the **UPy‐IKVAV**‐containing supramolecular solutions (c≈50–250 µm) (Figure 1C–E) and hydrogels (c≈4–18 mm) (Figure 1F–H) before proceeding with organoid culture. In the dilute state, we investigated whether **UPy‐IKVAV** was co‐assembled with the monofunctional supramolecular fibers or that self‐sorting was observed, using a combination of ultraviolet–visible (UV–vis) and circular dichroism (CD) spectroscopy as well as through cryo transmission electron microscopy (cryoTEM) images (Figure 1C–E). The co‐assembled solutions in the dilute state contained a similar ratio between the components as used in the supramolecular hydrogels, i.e., a molar ratio of **M‐UPy**/**UPy‐IKVAV** = 90/10%.

In case of self‐sorting, in which the two supramolecular species co‐exist in the sample without interacting with each other (**M‐UPy** separately existing from **UPy‐IKVAV**), it is expected that the absorbance spectrum in UV–vis spectroscopy (Figure 1C) and the molar ellipticity in CD spectroscopy (Figure 1D) of the mixtures overlap with the sum of the spectra of the two homo‐assemblies (calculated based on molar average). In contrast, when an interaction between non‐functionalized **M‐UPy** and functionalized **UPy‐IKVAV** supramolecular monomers would occur, the spectrum of the mixture is expected to deviate from the calculated molar averaged spectrum. Especially, in the UV–vis measurement, it was clear that the mixed spectrum deviated significantly from the calculated averaged molar spectrum, thus suggesting that **M‐UPy** and **UPy‐IKVAV** interact and exist in a co‐assembled state rather than a self‐sorted state. However, this was less obvious in the CD spectrum (Figure 1D). CryoTEM imaging of the co‐assembled solution at 250 µm concentration revealed the presence of µmeter‐long fibers (Figure 1E) that appear similar to **M‐UPy** fibers, suggesting that the long fibrous assemblies are not perturbed by the incorporation of the quite bulky GKKG‐IKVAV group (Schemes S1 and S2, Supporting Information) of **UPy‐IKVAV**.

We then investigated the IKVAV‐containing supramolecular hydrogels at 1.2 w/v% concentration with 1 mm **UPy‐IKVAV** and a molar ratio between M/B UPy of 80/1 with rheology (Figure 1F–H). The IKVAV‐containing supramolecular hydrogel has a *G*’ of ≈1 kPa (Figure 1F,H). We then subjected the supramolecular hydrogel to 1000% strain to completely break the hydrogel, after which its recovery was followed. Complete and immediate self‐healing behavior was observed through re‐formation of the supramolecular bonds (Figure 1G). Stress relaxation tests performed with an applied strain of 7.5% revealed fast recovery with a stress‐relaxing half‐life time (τ_1/2_) of ≈1000 s and ≈50% relaxed stress within a 1000 s timeframe (Figure 1H). To compare, the cytosine‐based, stress‐relaxing hydrogels that supported intestinal organoid morphogenesis as used by the Lutolf group showed “only” ≈10% relaxed stress within a 1000 s timeframe.^[^ ^52^ ^]^ Importantly, both the immediate self‐healing behavior and the fast stress relaxation are hypothesized to facilitate the growth of stem cells into multicellular organoids through dissipation of tissue forces.^[^ ^52^ ^]^

### 2.4 Laminin‐Derived UPy‐IKVAV Crucial for Correct Organoid Polarity

To determine the effect of ligand type on organoid polarity, different bioactive UPys monomers were included as ligands in the supramolecular hydrogels. Next to the laminin‐derived **UPy‐IKVAV**, other minimal synthetic sequences derived from the natural ligands present on fibronectin and collagen were tested: **UPy‐cRGD** as fibronectin‐derived ligand and **UPy‐GFOGER** as collagen‐derived ligand (Figure S5, Supporting Information), all key substituents of the intestinal crypt in‐vivo.^[^ ^67^ ^]^ In addition, **UPy‐PHSRN** was tested in combination with **UPy‐cRGD** (Figure S5, Supporting Information). PHSRN and RGD are both present in fibronectin, spaced 3.7 nm apart.^[^ ^68^ ^]^ PHSRN is a synergistic sequence of RGD, where PHSRN interacts with integrin α5, such that RGD gets in the correct conformational position to interact with integrin β1, also proven as effective synergistic pair synthetically.^[^ ^68^, ^69^ ^]^ **UPy‐cRGD** ^[^ ^50^ ^]^ and **UPy‐GFOGER** ^[^ ^49^ ^]^ were synthesized as previously reported. **UPy‐PHSRN** was successfully synthesized using the procedures reported in the Supporting Information (Scheme S3, Supporting Information), giving pure **UPy‐PHSRN** as confirmed using LC‐MS with a 63% yield (Figures S3 and S4, Supporting Information).

Single stem cells were encapsulated inside the different UPy hydrogels and cultured for 5 days both in presence and absence of an integrin activating antibody (TS2/16). Importantly, fluorescence recovery after photobleaching (FRAP) experiments with fluorescently labeled TS2/16 revealed a fast recovery after bleaching a spot (Figure S6A,C, Supporting Information). Analysis of the leftover bleached spot showed quick fluorescence recovery of ≈6 s, indicating (close to) free diffusion of the antibody throughout the hydrogels (Figure S6B,D, Supporting Information). This also overlaps with previous diffusion experiments using FITC‐dextran macromolecules with molecular weights of up to 2000 kDa (stokes radius ≈27 nm), revealing quick fluorescence recovery and thus free diffusion.^[^ ^50^ ^]^ Together, this indicates that the used integrin antibodies could freely diffuse through both the 1.2 and 2.5 w/v% UPy hydrogels. This is desired, because in this way the activating integrin antibodies are able to reach all cells and organoids inside the hydrogels.

**Figure 2:**
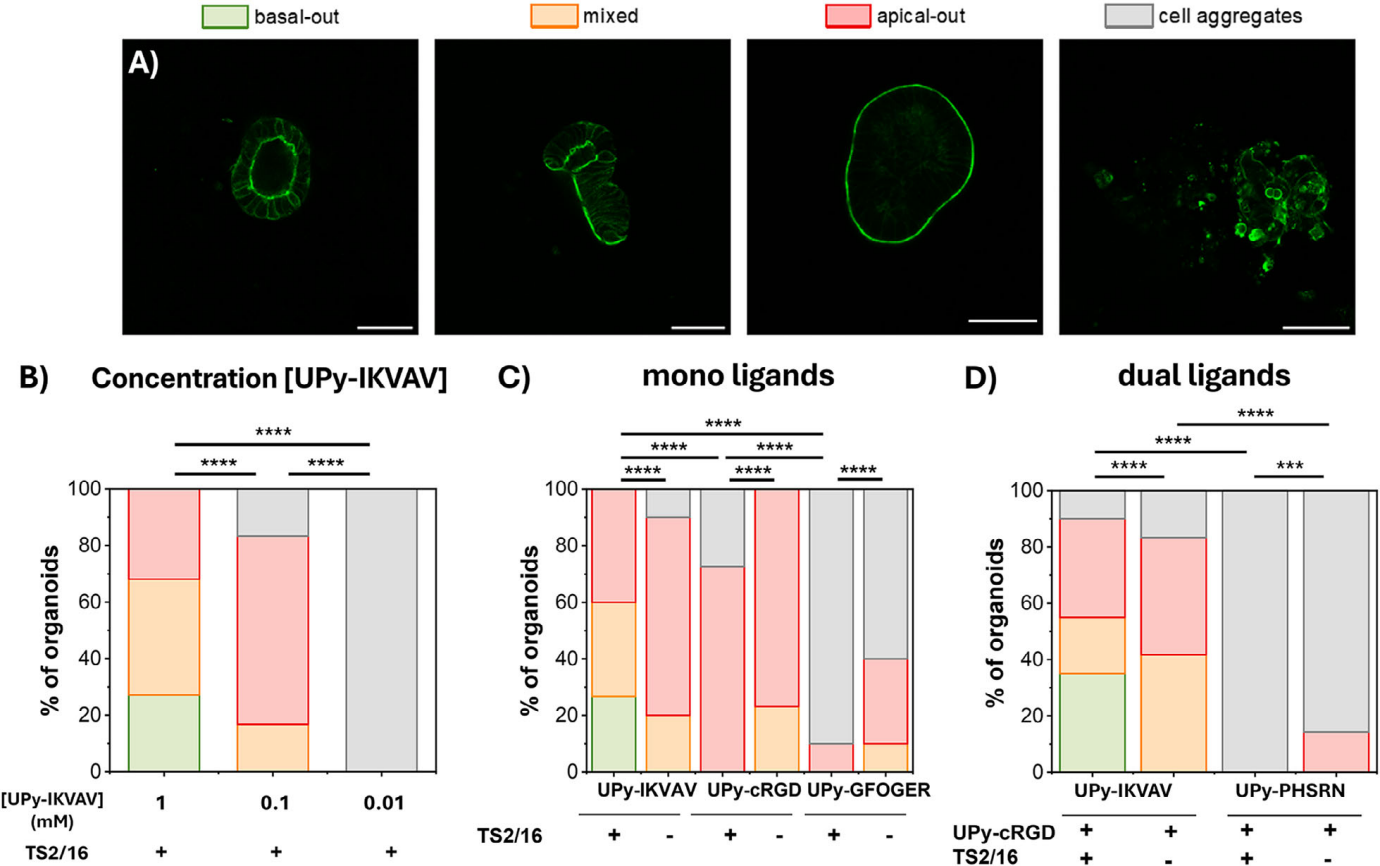
The influence of ligand type and concentration on human intestinal organoid polarity, using a fixed UPy concentration of 1.2 w/v% (*G*’ ≈1 kPa) and a molar ratio of M/B UPy = 80/1 (τ_1/2_ ≈1000 s). A) Confocal images of intestinal organoids cultured for 5 days inside UPy hydrogels, classified according to polarity of the formed cellular structures. B–D) Quantification of the frequencies of the different organoids that were formed inside 1.2 w/v% UPy hydrogels according to their polarity as influence of ligand type, with in B) the influence of UPy‐IKVAV concentration using 1, 0.1, and 0.01 mm UPy‐IKVAV, in C) with 1 mm of mono ligands UPy‐cRGD, UPy‐GFOGER or UPy‐IKVAV, in the presence and absence of TS2/16, and in D) with dual ligands containing 1 mm of each ligand, where UPy‐cRGD was always present and combined with UPy‐IKVAV or UPy‐PHSRN. In green organoids with correct, basal‐out polarity, in orange mixed polarity, in red reversed, apical‐out polarity, and in grey cellular aggregates. Green is F‐actin, an apical marker. Scale bar = 50 µm. The categorical organoid polarity data was subjected to a 𝜒2 test by comparing the groups individually to each other. *n* = 13–30 organoids per condition. Differences were considered as statistically significant when *p* < 0.05, with ^*^ *p* < 0.05; ^**^ *p* < 0.01; ^***^ *p* <0.001; ^****^ *p* < 0.0001.

After 5 days of culture, different organoids were formed and these were classified according to their polarity (**Figure** **2**A), with in green the correct, basal‐out polarity, in orange mixed polarity, in red inverted, apical‐out polarity and in gray cell aggregates. As positive control, intestinal organoids were also cultured in BME, which resulted in > 70% of intestinal organoids with correct basal‐out polarity (Figure S7, Supporting Information), and in presence with the integrin activating antibody (TS2/16) in even 100% correct basal‐out polarity (Figure 5B). As part of the characterization, immunofluorescent stainings of extracellular laminin were performed, confirming the presence of extracellular laminin (Figure S8, Supporting Information). It should be noted that the presence of extracellular laminin could be because 1) it is a component in BME and/or 2) because of laminin production and secretion by the intestinal organoids themselves – which is known to happen and to be detectable after already 3 days of culture in synthetic gels.^[^ ^70^, ^71^ ^]^

Only in the presence of 1 mm of laminin‐derived **UPy‐IKVAV** and the integrin activating antibody TS2/16, cells could grow into correctly polarized organoids (Figure 2A–C; Table S1, Supporting Information). Decreasing the **UPy‐IKVAV** concentration from 1 mm to 0.1, and finally to 0.01 mm resulted in the loss of correctly polarized organoids (Figure 2B; Table S3, Supporting Information). This demonstrates the crucial role of the laminin‐derived IKVAV in achieving correct basol‐out intestinal organoid polarity. Looking at the other ligand types, culturing the organoids in presence of fibronectin‐derived **UPy‐cRGD** resulted mostly in organoids with the wrong, apical‐out polarity (Figure 2C; Table S1, Supporting Information), failing to support correct organoid polarity. Cells cultured in the presence of the collagen‐derived **UPy‐GFOGER** were even worse and only led to cell aggregate formation (Figure 2C; Table S1, Supporting Information), while cells cultured in native, full‐length collagen could grow into correctly polarized organoids.^[^ ^30^, ^31^, ^32^, ^33^, ^37^ ^]^ GFOGER is known to mimic the integrin‐binding domain of native collagen, as it inhibits native collagen in binding to integrins when it is bound itself.^[^ ^62^ ^]^ Crucial concerns to achieve efficient GFOGER‐integrin interactions might include 1) the lack of a correct, helical confirmation of **UPy‐GFOGER**, which is also present in collagen, and 2) the need of a proper incorporation and availability for binding of this relatively big **UPy‐GFOGER** additive inside the monofunctional UPy stacks, which were both confirmed previously.^[^ ^72^, ^73^, ^74^ ^]^ Additional concerns include the fact that supraphysiological ligand concentrations are used inside our supramolecular hydrogels with 1 mm **UPy‐GFOGER** as compared to nm concentrations of collagen in the native ECM (equilibrium dissociation constant (K_d_) ≈nm range).^[^ ^75^ ^]^ Such supraphysiological ligand concentrations might lead to steric hindrance, as it is well known that high local ligand densities might render ligands unavailable for cell binding.^[^ ^76^ ^]^ In conclusion, we like to stress the crucial role of **UPy‐IKVAV** to achieve correct intestinal organoid polarity, as demonstrated by our results that 1) our other peptides (**UPy‐cRGD** and **UPy‐GFOGER**) did not result in correct basal‐out, organoid polarity, and 2) lowering the **UPy‐IKVAV** concentrations, from 1 to 0.1 to 0.01 mm, resulted in loss of correct basal‐out organoid polarity as well.^[^ ^49^ ^]^

Additional experiments with dual ligands were conducted to investigate possible synergistic effects between different ligands. Therefore, **UPy‐cRGD** (c = 1 mm) was always added and additionally, the fibronectin‐derived and synergistic sequence to RGD, **UPy‐PHSRN** (c = 1 mm), or the laminin‐derived sequence **UPy‐IKVAV** (c = 1 mm) were added (Figure 2D; Table S2, Supporting Information). Importantly, cell‐material interactions are not only influenced by ligand type and concentration, but also by ligand spacings. Especially for the synergistic RGD and PHSRN pair, both derived from fibronectin and spaced 3.7 nm apart in nature with ≈1 nm free movement of RGD.^[^ ^68^ ^]^ The synergistic effects between RGD and PHSRN have also been successfully mimicked before in a fully synthetic fashion, highlighting the crucial role of ligand spacings between the two ligands.^[^ ^69^ ^]^ In previous studies, we have done calculations on ligand spacings along the supramolecular fiber.^[^ ^40^, ^41^ ^]^ In this experiment using **UPy‐cRGD** and **UPy‐PHSRN** as dual ligands, a 1.2 w/v% gel with a ligand concentrations of 1 mm **UPy‐cRGD** with 1 mm **UPy‐PHSRN** was used, corresponding to concentrations of **M‐UPy** / **B‐UPy** / **UPy‐cRGD** / **UPy‐PHSRN** of 8.47 / 1.27 / 1.72 / 1.79 mg mL^−1^, equaling molar concentrations of 7.1 / 0.115 / 1 / 1 mm, and a molar ratio of 61 / 1 / 9 / 9, respectively (Table S2, Supporting Information). This results in effective ligand concentrations of each of the ligands of 11 mol% **UPy‐cRGD** and 11 mol% **UPy‐PHSRN** (Table S2, Supporting Information).^[^ ^40^ ^]^ Assuming a homogeneous distribution of the ligand‐functionalized UPys over the supramolecular fiber (assuming no clustering as a result of supramolecular interactions between the ligands, which might lead to self‐sorting and aggregation of the ligands), and a monomer‐to‐monomer distance of ≈0.5 nm,^[^ ^77^ ^]^ a ligand spacing of 1 ligand per ≈2.5 nm fibers for single, dimerized fibers and of 1 ligand per ≈0.9 nm fibers for a bundled stack consisting of three fibers^[^ ^78^ ^]^ can be determined for the 1.2 w/v% gels for each ligand. Since the supramolecular fibers are dynamic, the ligands should be spaced in a “close” enough distance to match the ideal distance between RGD and PHSRN of 3.7 ± 1 nm.

No synergistic effect could be detected, as the combination of **UPy‐cRGD** with **UPy‐IKVAV** in the presence of TS2/16 resulted in a similar fraction of correctly polarized organoids (≈25%) as to **UPy‐IKVAV** alone. While the addition of **UPy‐PHSRN** to **UPy‐cRGD** resulted only in the formation of cell aggregates, in contrast to organoids with inverted polarity for **UPy‐cRGD**.

### 2.5 Base Level of Stiffness Required to Offer Mechanical Support to Intestinal Organoids

Next, the influence of hydrogel stiffness on intestinal organoid polarity was investigated. Owing to the supramolecular nature of our hydrogel system, the hydrogel stiffness could be tuned independently from other hydrogel properties (ligand type and dynamics). Gel stiffness was tuned by varying the total w/v% of the hydrogels, while using a fixed absolute ligand concentration of 1 mm **UPy‐IKVAV** as well as a fixed molar ratio of M/B UPy of 80/1 to not alter dynamics (Table S4, Supporting Information).

**Figure 3:**
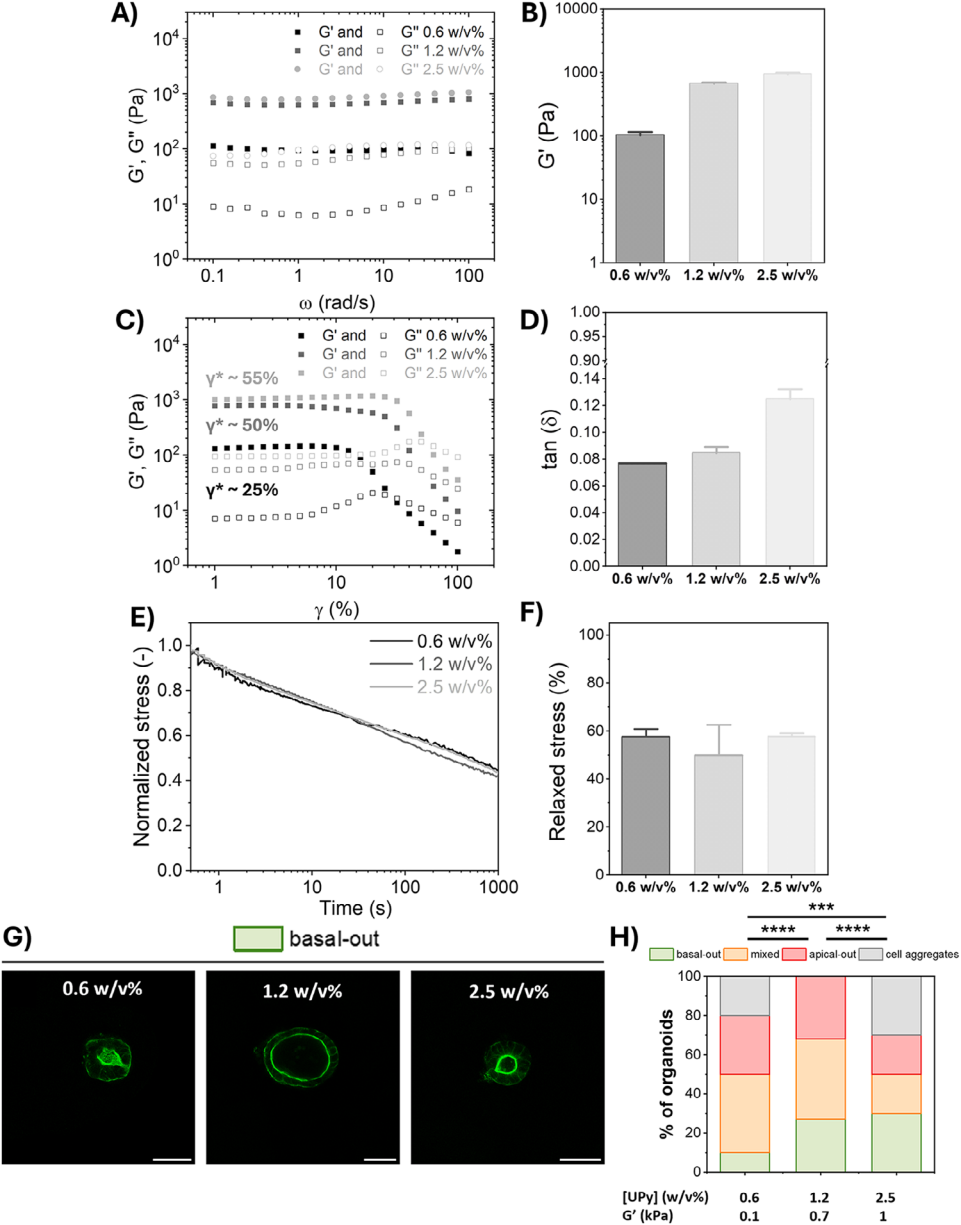
The influence of hydrogel stiffness on intestinal organoid polarity. A–F) Mechanical and dynamic characterization of UPy hydrogels upon changing the total UPy concentration, while keeping a fixed concentration of 1 mm **UPy‐IKVAV** and a fixed molar ratio of M/B = 80/1. In (A) a representative angular frequency sweep plot at 1% strain, in (B) G' quantified, in (C) a strain sweep plot measured at 1 rad s^−1^ both showing the storage (*G*′) and loss modulus (*G*″), in (D) the loss factor (tan(δ)) showing the ratio of viscous to elastic character, in (E) stress relaxation plot measured at 7.5% strain and a strain rise time of 0.01 s and in (F) the released stress quantified after 1000 s. (G) Representative fluorescence confocal images of intestinal organoids cultured for 5 days inside UPy hydrogels with 1 mm **UPy‐IKVAV** as ligand with varying stiffness. In green organoids with correct, basal‐out polarity, in orange mixed polarity, in red reversed, apical‐out polarity and in gray cellular aggregates. Green is F‐actin, an apical marker. Scale bar = 50 µm. H) Quantification of the frequencies of the different organoids that were formed inside UPy hydrogels according to their polarity as influence of hydrogel stiffness with 1 mM **UPy‐IKVAV** as ligand, in the presence of TS2/16. The categorical organoid polarity data was subjected to a 𝜒2 test by comparing the groups individually to each other. *n* = 13–30 organoids per condition. Differences were considered as statistically significant when *p* < 0.05, with ^*^ *p* < 0.05; ^**^ *p* < 0.01; ^***^ *p* < 0.001; ^****^ *p* < 0.0001. Quantified data is plotted with mean and standard error of the mean (SEM).

Rheological experiments revealed that while doing so, the stiffness of these hydrogels increased from *G*’ ≈ 0.1 to 0.7, and finally to 1 kPa upon increasing the UPy hydrogel concentration from 0.6, to 1.2 and to 2.5 w/v%, respectively (**Figure** **3**A,B). While doing so, the tan(δ), indicating the viscous‐to‐elastic response, was ≈0.1 for the 0.6 and 1.2 w/v% hydrogels, but increased to ≈0.2 for the 2.5 w/v% hydrogels, indicating a higher viscous‐like character for the 2.5 w/v% hydrogels and similar viscoelasticity for the 0.6 and 1.2 w/v% hydrogels (Figure 3D). We hypothesize that the highly dynamic character of **B‐UPy**, whose concentration is highest in the 2.5 w/v% hydrogels ([**B‐UPy**] = 2.54 mg mL^−1^ in 2.5 w/v% hydrogel versus 0.64 mg mL^−1^ in 0.6 w/v% hydrogel, Table S4, Supporting Information), is responsible for this observation. Interestingly, this tan(δ) seemed to match with the strain at which the hydrogels break (γ^*^), being ≈25%, 50% and 55% for the 0.6, 1.2 and 2.5 w/v% hydrogels, respectively (Figure 3C,D). This shows that upon increasing the w/v% of the hydrogels, and hence the **B‐UPy** concentration, the hydrogels become more viscous‐like, likely due to the higher PEG content (Figure 3C). To investigate the hydrogel dynamic behavior, stress relaxation experiments were conducted. A strain of 7.5% was applied to the hydrogels, after which the ability of the hydrogels to dissipate the applied stress was followed over time for 1000 s. Importantly, all hydrogels displayed similar stress relaxation, with ≈50% of the stress released after 1000 s, corresponding to a τ_1/2_ of ≈1000 s. So, while changing the hydrogel stiffness, the bulk dynamics were not altered (Figure 3E,F).

To study the influence of hydrogel stiffness on the intestinal organoid polarity, cells were encapsulated in **UPy‐IKVAV**‐containing hydrogels with increasing stiffness from *G*’ ≈ 0.1 to 0.7 and to 1 kPa for the 0.6, 1.2 and 2.5 w/v%, respectively. For the soft, 0.1 kPa hydrogels, a low fraction of correctly polarized organoids (≈10%) was observed, while increasing hydrogel stiffness toward 1 kPa yielded a higher fraction of correctly polarized organoids (≈30%, Figure 3G,H). This shows that a base level of stiffness (≈0.7 kPa) is required to offer enough mechanical support to the cells. Besides, the softest (0.1 kPa) and stiffest hydrogels (1 kPa) yielded smaller organoids than the 1.2 w/v% hydrogels with *G*’ ≈0.7 kPa, which seemed to be an optimum (Figure 3G,H). Together, these results show that a base level of stiffness is required to offer enough mechanical support, next to the crucial role of ligand type, to support correct intestinal organoid polarity.

### 2.6 Hydrogel Dynamics Further Support Correct Intestinal Organoid Polarity

Next, by supramolecular tuning, we investigate the influence of hydrogel dynamics on intestinal organoid polarity through supramolecular tuning. To tune hydrogel dynamics, the ratio of **M‐UPy** to **B‐UPy** was varied by increasing the content of the inherently dynamic **B‐UPy** from 80/1 to 9/1 (in mol), while a fixed absolute ligand concentration of 1 mm **UPy‐IKVAV** was used, as well as a fixed total UPy hydrogel concentration of 1.2 w/v% to not alter stiffness (Table S5, Supporting Information).

**Figure 4:**
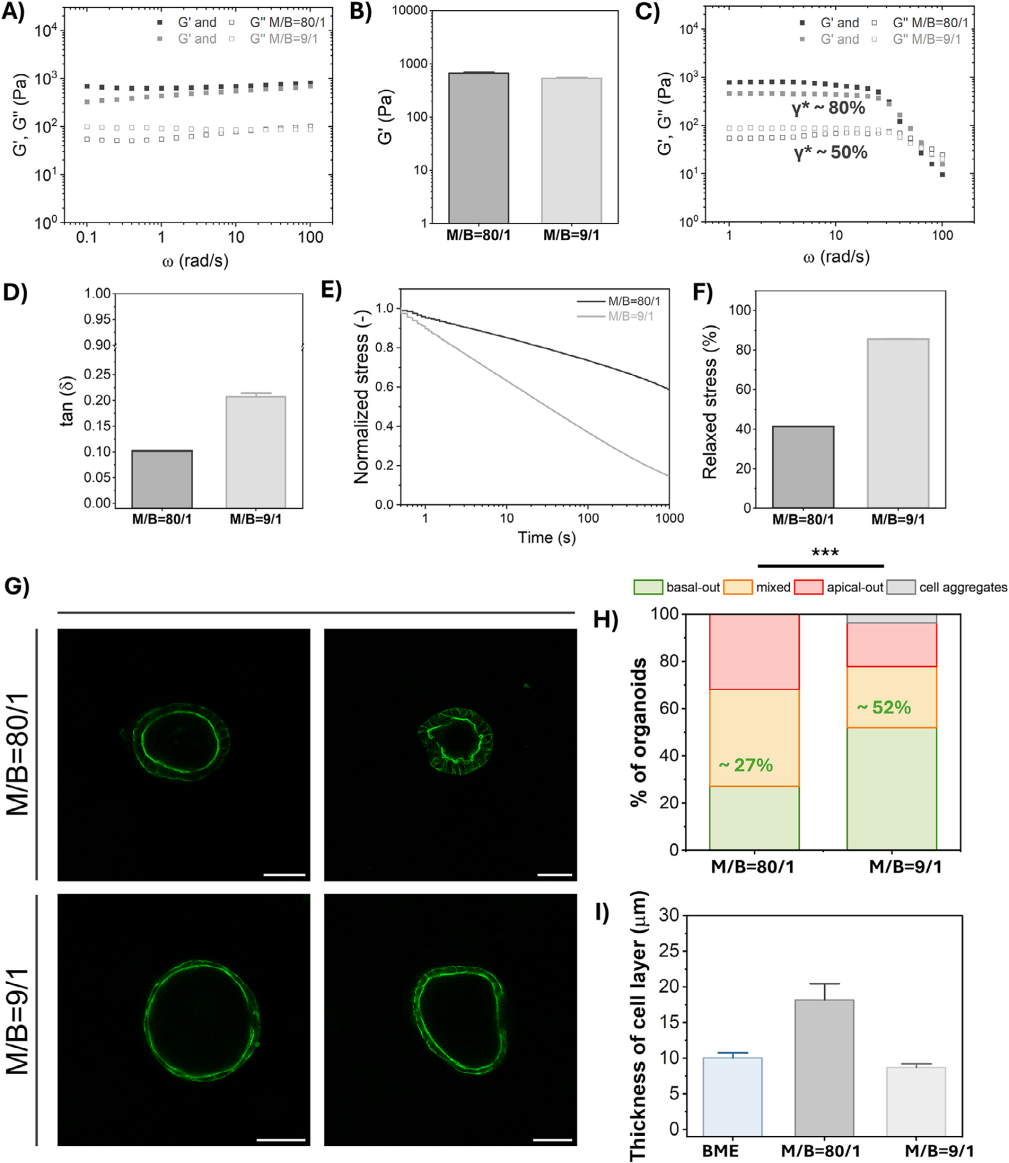
The influence of hydrogel dynamics on intestinal organoids polarity. A–F) Mechanical and dynamic characterization of UPy hydrogels upon changing the M/B UPy molar ratio, while keeping a fixed concentration of 1 mm **UPy‐IKVAV** and a fixed total UPy concentration of 1.2 w/v%. (A) A representative angular frequency sweep plot at 1% strain, in (B) *G*’ quantified, in (C) a strain sweep plot measured at 1 rad s^−1^ showing the storage (*G*’) and loss modulus (*G*”), in (D) the loss factor (tan(**δ**)) showing the ratio of viscous to elastic character, in (E) stress relaxation plot measured at 7.5% strain and a strain rise time of 0.01 s and in (F) the released stress quantified after 1000 s. G) Representative fluorescence confocal images of intestinal organoids cultured for 5 days inside the normal (**M/B = 80/1**, top) and dynamic (**M/B = 9/1**, bottom) 1.2 w/v% UPy hydrogels (*G*’ ≈0.7 kPa) and with 1 mm **UPy‐IKVAV** as ligand and in presence of TS2/16 integrin activating antibody. In green organoids with correct, basal‐out polarity, in orange mixed polarity, in red reversed, apical‐out polarity and in grey cellular aggregates. Green is F‐actin, an apical marker. Scale bar = 50 µm. (H) Quantification of the frequencies of the different organoids that were formed according to their polarity. I) Quantification of cell layer thickness in µm. The categorical organoid polarity data was subjected to a 𝜒2 test by comparing the groups individually to each other. *n* = 13–30 organoids per condition. Differences were considered as statistically significant when *p* < 0.05, with ^*^ *p* < 0.05; ^**^ *p* < 0.01; ^***^ *p* < 0.001; ^****^ *p* < 0.0001. Quantified data is plotted with mean and SEM.

Rheological experiments showed that hydrogel stiffness indeed remained roughly the same with a *G*’ ≈ 0.7 kPa for **M/B = 80/1** and ≈0.6 kPa for the **M/B = 9/1** hydrogels (**Figure** **4**A–C). The tan(δ), indicating the viscous‐elastic response, was ≈0.1 for the **M/B = 80/1** hydrogels and ≈0.2 for the **M/B = 9/1** hydrogels, showing a more viscous character for the **M/B = 9/1** hydrogels (Figure 4D). Moreover, the **M/B = 9/1** hydrogels could handle more stain before rupture (γ^*^ ≈ 80%) as compared to the regular **M/B = 80/1** hydrogels, which broke already at 50% applied strain and were thus more brittle (Figure 4C). Interestingly, this higher viscous‐like character for the **M/B = 9/1** hydrogels, which contained a higher content of the dynamic **B‐UPy** with PEG10k (as compared to the **M/B = 80/1** hydrogels) is also consistent with the higher viscous behavior for the stiffest, 2.5 w/v% hydrogels, whose **B‐UPy** content is also highest as well (as compared to the softer hydrogels with lower **B‐UPy** concentrations). With respect to bulk dynamics, the **M/B = 80/1** hydrogels relaxed ≈45% of the stress, while the **M/B = 9/1** hydrogels dissipated ≈85% within the measured 1000 s timeframe, corresponding to a stress‐relaxing half‐life time of ≈1000 s for the **M/B = 80/1** hydrogel and of ≈30 s for the **M/B = 9/1** hydrogel (Figure 4E,F). Together, this indicates that the **M/B = 9/1** exhibited faster bulk dynamics with faster fiber rearrangements than the **M/B = 80/1** hydrogel. This initially might seem counterintuitive, as more cross‐links usually strengthen networks and slow down dynamics in covalent networks.^[^ ^79^ ^]^ However, in our supramolecular UPy hydrogels, the **B‐UPy** containing a long PEG 10k chain is highly dynamic. Thus, despite the increased number of (supramolecular) cross‐links, it leads to a more dynamic network.

To study the influence of hydrogel dynamics on intestinal organoid polarity, cells were encapsulated in previously mentioned hydrogels containing a ratio of **M/B = 80/1** versus in more dynamic hydrogels with **M/B = 9/1**. Interestingly, a higher number of correctly polarized organoids is observed in the more dynamic **M/B = 9/1** hydrogels (≈52%) than in the regular **M/B = 80/1** hydrogels (≈27%) (Figure 4G,H). We hypothesize that the increased stress relaxation allows faster fiber rearrangements, supporting tissue growth from single cells into multicellular organoids. Interestingly, organoids cultured in the more dynamic hydrogels contained a thinner layer of (peripheral) cells than the organoids cultured in the regular hydrogels, roughly similar to organoids cultured in BME (Figure 4I; Figure S9, Supporting Information), which is beneficial as a thicker cell layer might indicate unwanted differentiation. However, quantification of the organoid diameter and size revealed no major differences between regular and dynamic hydrogels (Figure S9, Supporting Information). Both hydrogels contained organoids with a diameter of ≈80–90 µm and a size of ≈6000 µm^2^, which was smaller than organoids cultured in BME with ≈100 µm and a size of ≈8000 µm^2^ (Figure S9, Supporting Information). Altogether, when the correct ligand type is presented to the organoids, the increased hydrogel dynamics catalyzes intestinal organoid polarity.

### 2.7 Elucidating the IKVAV‐Organoid Interaction

**Figure 5:**
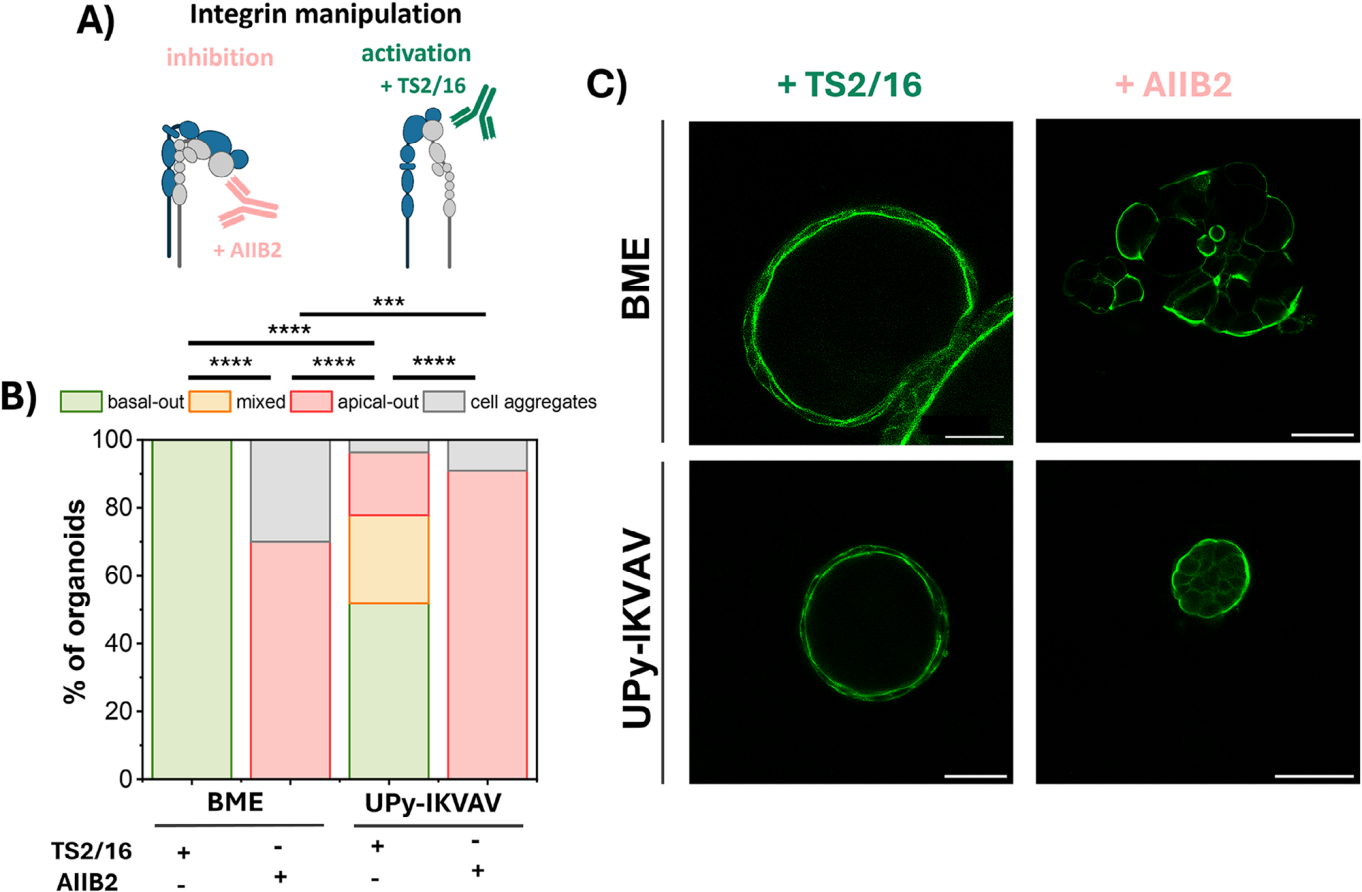
The IKVAV‐organoid interaction is integrin β1‐mediated. A) Aim of this experiment in cartoon style to investigate whether the IKVAV‐organoid interaction is integrin‐mediated, performed through manipulation of integrins with integrin‐activating (TS2/16) and inhibiting (AIIB2) antibodies. B) Quantification of the frequencies of the different organoids that were formed according to their polarity inside BME and the best performing **UPy‐IKVAV** hydrogel, in the presence of the activating TS2/16 or in presence of the integrin blocking AIIB2 antibody. C) Representative fluorescence confocal images of intestinal organoids cultured in BME or UPy with the integrin activating or inhibiting antibodies. The categorical organoid polarity data was subjected to a 𝜒2 test by comparing the groups individually to each other. *n* = 13–30 organoids per condition. Differences were considered as statistically significant when *p* < 0.05, with ^*^ *p* < 0.05; ^**^ *p* < 0.01; ^***^ *p* <0.001; ^****^ *p* < 0.0001. Green is F‐actin, an apical marker. Scale bar = 50 µm.

To elucidate the mechanism underlying the IKVAV‐organoid interactions and to study whether these interactions are integrin‐mediated, integrin activity was manipulated (**Figure** **5**A). Therefore, the best performing UPy hydrogel was chosen, i.e., 1.2 w/v% UPy hydrogel (*G*’ ≈ 1 kPa) with 1 mm **UPy‐IKVAV** and M/B = 9/1 (τ_1/2_ ≈ 30 s), referred to as **UPy‐IKVAV**. An integrin β1 blocking antibody (AIIB2) was used, known to reverse the polarity of enteroids cultured in BME to an apical‐out polarity.^[^ ^80^ ^]^ The integrin β1 blocking antibody was added to both the cell suspension inside the hydrogels and the culture medium that was added on top of the hydrogels, and compared to the regular situation in which the activating integrin antibody TS2/16 was present. For organoids cultured in BME, the addition of the integrin blocking antibody AIIB2 to the culture resulted in a loss of the correct organoid polarity, causing inverted apical‐out organoids (Figure 5B,C; Figure S7, Supporting Information). Similarly, the addition of the β1 integrin blocking antibody to **UPy‐IKVAV** hydrogels resulted in organoids with almost 100% inverted, apical‐out polarity (Figure 5B,C). This result suggests that the IKVAV‐organoid interaction is integrin β1‐mediated.

**Figure 6:**
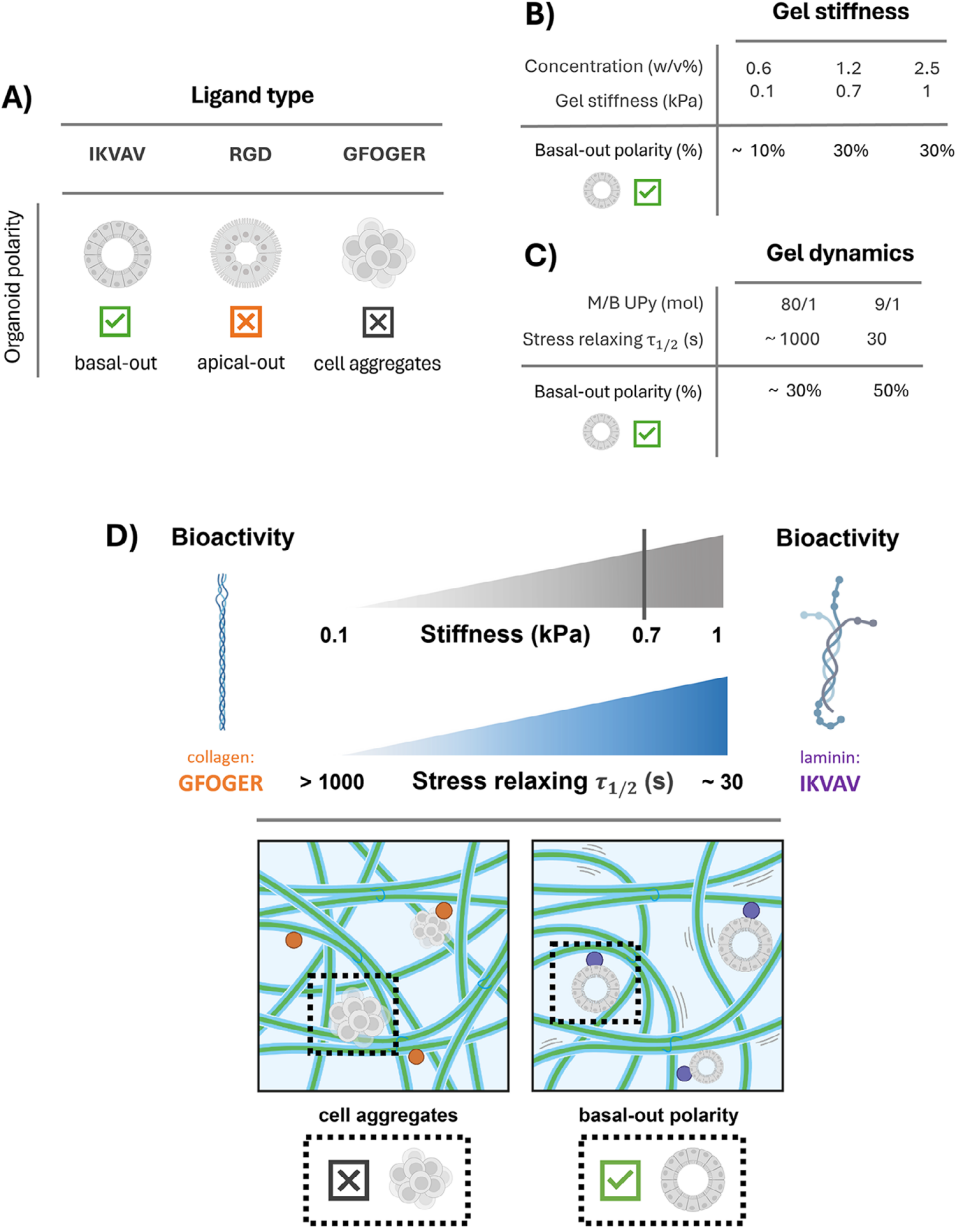
Summary of the main findings and conclusion, presenting design rules to control intestinal organoid polarity in completely synthetic hydrogels. A–C) Summary of the results, showing the influence of A) ligand type, B) gel stiffness and C) gel dynamics on intestinal organoid polarity. D) Conclusion of our work, showing the laminin‐derived IKVAV sequence is crucial for correct, basal‐out organoid polarity. Furthermore, a base level of stiffness is required (≈0.7 kPa) to offer mechanical support, while increased gel dynamics (τ_1/2_ ≈ 30 s) further supports correct intestinal organoid polarity.

In summary, we have created design rules to control intestinal organoid polarity in synthetic, supramolecular UPy‐based hydrogels (**Figure** **6**D). We revealed that the laminin‐derived IKVAV sequence can mimic basement membrane laminin and is crucial for achieving the correct, basal‐out polarity of intestinal organoids (Figure 6A), as the collagen‐derived **UPy‐GFOGER** as well as the fibronectin‐derived **UPy‐cRGD** resulted in cell aggregates and inverted, apical‐out organoids, respectively. Culture in soft, **UPy‐IKVAV** containing hydrogels (*G*’ ≈0.1 kPa) resulted in only a low fraction (≈10%) of correctly polarized organoids, while increasing the hydrogel stiffness from 0.1 to 0.7, and to 1 kPa further supported the growth of correctly polarized organoids (≈30%, Figure 6B). This shows that a base level of stiffness is crucial to provide mechanical support to the cells. Increasing the bulk dynamics with a stress relaxing τ_1/2_ from ≈1000 to 30 s further stimulated the growth of correctly polarized organoids (≈50%, Figure 6C). We speculate that the faster fiber rearrangements in the fast‐relaxing hydrogels facilitate the growth of single cells into multicellular organoids by allowing better dissipation of tissue exerted forces.

## 3 Conclusion

Design rules for controlling the polarity of human intestinal organoids were established using completely artificial, dynamic hydrogels. The laminin‐mimicking sequence IKVAV is crucial to establishing correct intestinal organoid polarity. Besides, intestinal organoids respond to gel stiffness, as a base level of stiffness (≈0.7 kPa) was required to offer mechanical support to the cells. Fast gel dynamics (stress‐relaxing τ_1/2_ ≈ 30 s) further catalyze the growth of correctly polarized intestinal organoids. Manipulation of integrins revealed that the IKVAV‐organoid interaction is integrin β1‐mediated.

With these findings, we highlight the unique advantages and potential of employing supramolecular hydrogels as ECM mimics to control cell and organoid culture in a synthetically controlled environment. We emphasize the crucial role of and the complex interplay between matrix bioactivity, mechanics, and dynamics to achieve the correct organoid polarity. Future work may utilize these fundamental design rules to culture more complex living tissues in a fully controlled, synthetic fashion, useful for (personalized) drug screening and tissue replacements applications.

## 4 Experimental Section

### Analytical Liquid Chromatography‐Mass Spectrometry (LC‐MS)

Purity and exact mass of the synthesized peptides and peptide‐functionalized UPys was determined using a LCQ Fleet (Thermo Finnigan) ion‐trap mass spectrometer in ESI mode equipped with a Shimadzu Autosampler (SIL 20‐AC xr) and Shimadzu PDA detector (SPD‐M20A). Solvents were pumped with a flow of 0.3 mL min^−1^ using a high‐pressure gradient system using two LC‐20‐AD xr pumps (Shimadzu). Before mass analysis, the crude was run over a reversed phase C18 column (Kinetex EVO, 5 µm, 2.1 × 50 mm, Phenomenex) using a 5–90 v/v% acetonitrile linear gradient in water with 0.1 v/v% formic acid.

### UV–Vis Spectroscopy

UV‐vis spectroscopy was performed at a final concentration of 50 µm, of which 10 mol.% **UPy‐IKVAV** in the mixtures. UV–vis spectroscopy was measured on an Agilent Cary 3500 Multicell UV–vis spectrophotometer using a Multicell Peltier UV–vis detector. Measurements were performed at 20 °C in quartz cuvettes with a pathlength of 1 cm and a UPy concentration of 50 µm. For all samples, a baseline of the corresponding solvent (PBS pH 7.4) was measured. All measurements were recorded with a bandwidth of 1.0 nm, a scan speed of 300 nm min^−1^, and a data interval of 0.1 nm, spanning the UV–vis range of 350–200 nm.

### CD Measurements

CD spectra were measured at a final concentration of 50 µm, of which 10 mol.% **UPy‐IKVAV** in the mixtures. CD spectra were measured on a JASCO J‐815 CD Spectrometer equipped with a Peltier temperature controller. Measurements were performed at 20 °C in quartz cuvettes with a pathlength of 1 cm and a UPy concentration of 50 µm. A scanning rate of 100 nm min^−1^, a bandwidth for monitoring of 1.0 nm, a response time of 1 s, a data pitch of 0.1 nm, triple accumulation and a scanning range of 350–200 nm were employed.

### CryoTEM

CryoTEM images were created at a final concentration of 250 µm, of which 10 mol.% **UPy‐IKVAV** in the mixtures. Vitrified thin films for CryoTEM analysis were prepared using an automated vitrification robot (FEI Vitrobot Mark IV) by plunge vitrification in liquid ethane. Before vitrification, a 200‐mesh copper grid covered with a Quantifoil R 2/2 holey carbon film (Quantifoil Micro Tools GmbH) was surface plasma treated for 40 s using a Cressington 208 carbon coater. CryoTEM imaging was carried out on the Glacios (Thermo Fisher), equipped with a field emission gun (X‐FEG), Ceta 16 m camera and a Falcon 4i direct electron detector. The microscope was operated at 200 kV acceleration voltage in bright‐field TEM at a nominal magnification of 6.500 × and a dose rate of 2 e^−^ Å^−2^·s; or at 24.000× magnification and a dose rate of 4 e^−^ Å^−2^·s; both with a 1s image acquisition time.

### Rheology

Rheological measurements were carried out on an Anton Paar rheometer (MCR 501). Hydrogels were prepared according to the supramolecular formulation method and scooped onto the rheometer. Supramolecular hydrogels were measured using a cone plate geometry with a diameter of 8 mm and a gap height of 49 µm at room‐temperature. To minimize sample drying during measurements, a solvent trap with water was used. Frequency sweep experiments were recorded from ω = 0.1–100 rad s^−1^ at a constant strain of γ = 1%. Strain sweep experiments were recorded from γ = 1–100% at a constant frequency ω of 1 rad s^−1^. Stress relaxation tests were performed by applying a strain of γ = 7.5% and a constant frequency ω of 1 rad s^−1^. For self‐healing tests, the supramolecular hydrogel was subjected to the following cycle: ω = 1 rad s^−1^ and γ = 1% for 60 s as “rested state,” followed by a “stressed state” to break the supramolecular hydrogel using ω = 1 rad s^−1^ and γ = 1000% for 60 s, after which its recovery was followed up to 10 min using ω = 1 rad s^−1^ and γ = 1%.

### Supramolecular Hydrogel Formulation of Other Bioactive UPys

**M‐UPy** and bioactive UPy molecules (i.e., **UPy‐cRGD**, **UPy‐PHSRN**, **UPy‐GFOGER**) were both dissolved under basic conditions and elevated temperature (70 °C) at the desired stock concentration. Bioactive UPys were then combined with **M‐UPy** under basic conditions to ensure the homogeneous distribution of bioactive UPys. The bioactive mixture was then neutralized using an adequate amount of 2 m HCl in MQ water. A slightly different procedure was used for **UPy‐PHSRN**, which was first neutralized (as well as **M‐UPy**) to prevent crashing out of solution, after which it was combined under neutral conditions with **M‐UPy** solution. Thereafter, the exact same procedure for formulating the supramolecular hydrogels as well as for stem cell encapsulation was used as described in the main text in section “2.2 Formulation of IKVAV‐containing supramolecular fibers and hydrogels.”

### Fluorescence Recovery After Photobleaching (FRAP)

FRAP measurements were carried out using a Leica TCS SP5 inverted confocal microscope (Leica Microsystems) equipped with a 20× objective (HCX PL APO CS 20.0 × 0.70 DRY UV) or a 40× objective (HCX PL APO CS 40.0 × 1.1 water UV). TS2/16 antibody conjugated with Alexa647 was used as a model system to study the diffusion capability of the integrin‐activating (TS2/16) and inhibiting (AIIB2) antibodies throughout the supramolecular UPy hydrogel. TS2/16‐Alexa647 was dissolved in 1x HBS with 20 mm HEPES with pH 7.3, and 500 mm NaCl. Non‐functionalized UPy hydrogels (i.e., without **UPy‐IKVAV**) were prepared as described in section 2.2, which describes the supramolecular hydrogel formulation procedure. TS2/16‐Alexa647 antibody and non‐functionalized UPy hydrogel were mixed in a 1:1 volume ratio. The final concentration of TS2/16‐Alexa647 antibody was 0.5 mg/mL. The final concentration of UPy hydrogel was either 1.2 or 2.5 w/v%. The samples, each containing 0.5 mg mL^−1^ of TS2/16‐Alexa647 antibody, were prepared into an Ibidi micro‐angiogenesis glass‐bottom well plate and stored vial at 4 °C overnight. To minimize sample drying during the measurements, the empty wells were filled with water. Exchange dynamics experiments were carried out via sample illumination using a white laser at λ = 633 nm for Cy5 excitation. Emission was collected at λ = 645–735 nm using a hybrid detector. A circular area with a diameter of 20 or 50 µm was photo‐bleached at 98% laser power for 200–300 frames (0.653 frame s^−1^), and the post‐bleaching time‐lapse imaging was performed for a maximum 6 h, depending on the samples. To correct for the drift of the hydrogel, the region of interest was adjusted manually. Image analysis was performed in Leica AF software, and data processing in Origin. Data was normalized by dividing the fluorescence intensity by the maximum observed fluorescence intensity in a non‐bleached circular area of the same size for each measurement point. The fluorescent recovery data points were fitted using a single exponential growth model.

### Organoid Medium

*Base medium* for washing consisted of Advanced DMEM/F12 Reduced Serum (Gibco) supplemented with 1 v/v% penicillin‐streptomycin (P/S), 1 v/v% GlutaMAX and 1 v/v% HEPES.

*Human colon medium* consisted of the base F12 medium supplemented with additional Next‐generation Surrogate Wnt,^[^ ^81^ ^]^ R‐spondin‐1, B27, Nogging, EGF, A83 (a TGF‐β inhibitor), prostaglandin E2 (PGE2), N‐acetyl‐L‐cysteine (NAC), nicotinamide (NIC) and p38 mitogen‐activated protein kinase (MAPK) inhibitor.

### Organoid Culture

Healthy, adult, patient‐derived p26N stem cells were normally cultured in BME using colon medium in a 6‐ or 12‐well plate until sufficient organoids were grown. Culture plates were incubated at 37 °C in 5% CO_2_ one day prior to adding the p26N cells. To harvest the organoids, organoids were treated with ≈1 mL cell recovery solution per well in a 6‐well plate for 3 min. Organoids were removed from the plate by manually scraping them using a P1000 pipette and subsequently pipetting the solution off the plate in a 15 mL Falcon tube. The organoids (still embedded in BME) were placed on ice for ≈20–30 min to obtain a more liquid‐like state of BME. Then, organoids were pelleted by centrifuging at 1500 rpm for 3 min at 4 °C, after which the suspension was removed. Organoids were resuspended in washing medium and pelleted again by centrifuging at 1500 rpm for 3 min at 4 °C. The suspension was removed and organoids were then resuspended in a 2:1 (v/v%) ratio of TrypLE: washing medium containing Rho kinase (ROCK) inhibitor (1:1000) to break down the organoids toward single cells for ≈20 min at 37 °C. This process was followed using a brightfield microscope. Once the organoids were dissociated, the suspension was pelleted again by centrifuging at 1500 rpm for 3 min. The supernatant was discarded and p26N cells were now resuspended in colon medium, which were subsequently encapsulated inside UPy hydrogels, as described in Section 2.2.

### Immunofluorescence Staining and Confocal Microscopy

After organoid culture for 5 days, the samples were washed with PBS, followed by fixation for 10 min with 3.7 v/v% formaldehyde in PBS and washing with PBS twice afterward. Afterwards, samples were stained with phalloidin to visualize F‐actin (1:400, apical marker) in PBS with 0.2 v/v% Triton X‐100 in the dark at room‐temperature for 1 h. Finally, the samples were washed with PBS containing 0.2 v/v% Triton X‐100. For the laminin stainings, the samples were first blocked with 5 v/v% FBS in PBS at room‐temperature for 1 h, and afterwards washed three times with PBS. The samples were then stained with anti‐laminin γ‐1 antibody (Abcam, ab17792, 1:100 dilution in PBS) at room‐temperature for 1 h, and afterwards washed three times with PBS. The sample was then incubated with anti‐rat secondary antibody containing Alexa647 (1:500 dilution in PBS) at room‐temperature for 30 min and afterward washed three times with PBS. Immediately thereafter, the samples were imaged (and only mounted, with or without DAPI, if necessary) on a Leica TCS SP5 inverted confocal microscope using 10× (HCX PL APO CS 10.0 × 0.4 DRY UV), 20× (HCX PL APO CS 20.0 × 0.7 DRY UV) and 40× (HCX PL APO CS 40.0 × 1.1 water UV) objectives. Image analysis and quantification of organoid polarity, shape, and size (*n* = 13–30 organoids per condition) was performed using LAS X and Fiji (ImageJ) software.

### Statistical Analysis

All quantified data are presented as the mean ± SEM. Image analysis and quantification of organoid polarity, shape, and size (*n* = 13–30 organoids per condition) was performed using LAS X and Fiji (ImageJ) software. Statistical analyses were performed using GraphPad Prism 8.0.2 (La Jolla, CA, USA). Stress relaxation data is presented in a normalized fashion, where data is presented relative to the relaxed stress at t = 0.5 s (i.e., containing the highest *G*’ (Pa) and the least amount of relaxed stress). Biological data concerning the categorical organoid polarity data were subjected to 𝜒2 test by comparing the groups individually to each other. *n* = 13–30 organoids per condition. Differences were considered as statistically significant when p < 0.05, with *p < 0.05; ^**^ *p* < 0.01; ^***^ *p* <0.001; ^****^ *p* < 0.0001.

## Conflict of Interest

Patricia Dankers is co‐founder and shareholder of spin‐off company VivArt‐X that focusses on women's health using supramolecular materials; especially targeting breast regeneration.

## Supporting information



Supporting Information

## Data Availability

The data that support the findings of this study are available from the corresponding author upon reasonable request.
