# Supplementary material for: Controlling Intestinal Organoid Polarity using Synthetic Dynamic Hydrogels Decorated with Laminin‐Derived IKVAV Peptides
Source: Adv Healthc Mater. 2025 Sep 4;15(8):e02079. doi: 10.1002/adhm.202502079 (PMC12927536; doi:10.1002/adhm.202502079)
Supplement: Supplementary file 1 — Supporting Information [file ADHM-15-0-s001.docx]

**Supporting information for**

Controlling Intestinal Organoid Polarity using Synthetic Dynamic Hydrogels Decorated with Laminin-Derived IKVAV Peptides

Laura Rijns^1,2^, Joost A. P. M. Wijnakker^3^, Victor A. Veenbrink^1,2^, Riccardo Bellan^1,2^, Fenna W. B. Craenmehr^1,2^, Simone I. S. Hendrikse^1,2^, Johnick F. van Sprang^1,2^, Wim de Lau^3^, E. W. Meijer^1,4,5^, Hans Clevers^3,6^, Patricia Y. W. Dankers^1,2,4 *^

^1^ Institute for Complex Molecular Systems, Eindhoven University of Technology, 5600 MB, Eindhoven, the Netherlands.

^2^ Department of Biomedical Engineering, Eindhoven University of Technology, 5600 MB, Eindhoven, the Netherlands.

^3^ Hubrecht Institute, University Medical Center (UMC) Utrecht, 3584CX, Utrecht, the Netherlands.

^4^ Department of Chemical Engineering and Chemistry, Eindhoven University of Technology, 5600 MB, Eindhoven, the Netherlands.

^5^ The School of Chemistry and the RNA Institute, University of New South Wales, Sydney, New South Wales 2052, Australia.

^6^ Current address: Pharma, Research and Early Development of F. Hoffmann-La Roche Ltd, 4070 Basel, Switzerland.

*corresponding author: [p.y.w.dankers@tue.nl](mailto:p.y.w.dankers@tue.nl)

**Materials and Methods**

**General**All reagents, chemicals, materials and solvents were obtained from commercial sources and were used as received. All solvents were of peptide synthesis grade quality. In the synthesis procedures, equivalents (eq) are molar equivalents. Peptides were synthesized using standard Fmoc chemistry unless stated otherwise.

1. **Synthesis and characterization of bioactive UPy**

**UPy-IKVAV synthesis**

All reactions were performed under high shear mixing using an overhead stirrer at 1000 rpm, 60 °C, unless stated otherwise. Rink amide MBHA resin (78.4 mg, 0.04 mmol, 1 eq) was swollen in 4 mL DMF for 1 hour at RT. The resin was deprotected 2 x 5 min with 20 v/v% piperidine in DMF. Each subsequent amino acid (0.08 mmol, 2 eq) was coupled once with O-(1H-6-Chlorobenzotriazole-1-yl)-1,1,3,3-tetramethyluronium hexafluorophosphate (33.1 mg, 0.08 mmol, 2 eq) and N-Ethyl-N-(propan-2-yl)propan-2-amine (DIPEA) (27.9 µL, 0.16 mmol, 4 eq) in 4 mL DMF and once with HATU (30.4 mg, 0.08 mmol, 2 eq) and 2,4,6-Trimethylpyridine (21 µL, 0.16 mmol, 4 eq) in 4 mL DMF. After this a colorimetric assay was performed on a small number of beads using 2 v/v% acetaldehyde in DMF and 2 w/v% 2,3,5,6-Tetrachlorocyclohexa-2,5-diene-1,4-dione in DMF for 5 min at RT. Coupling with HATU and 2,4,6-Trimethylpyridine (21 µL, 0.16 mmol, 4 eq) was repeated until the colorimetric assay yielded colorless beads. Following this, the deprotection, coupling and colorimetric assay were repeated for the next amino acid until the sequence of Fmoc-Gly-Lys(Boc)-Lys(Boc)-Gly-Ile-Lys(Boc)-Val-Ala-Val was achieved. The peptide on resin was deprotected 2 x 10 min with 20 v/v% piperidine in DMF. **UPy-C_6_-U-C_12_-Ur-PEG_12_-C_2_-TFP** (102.9 mg, 0.08 mmol, 2 eq) and DIPEA (55.7 µL, 0.32 mmol, 8 eq) were dissolved in 2 mL chloroform and 2 mL DMF. The reaction mixture was added to the peptide on resin and reacted overnight under shaking conditions at room temperature. The UPy-peptide was cleaved from the resin using a 96.5:2.5:1 v/v% TFA:H_2_O:TIS mixture. The cleavage mixture was precipitated in ice-cold diethyl ether. The precipitate was collected, dissolved in water and lyophilized yielding 41.2 mg **UPy-IKVAV** (51%) as a fluffy white solid.

**UPy-PHSRN synthesis**

Fmoc-Gly-Pro-His(Trt)-Ser(tBu)-Arg(Pbf)-Asn(Trt) (36.2 mg, 0.01 mmol, 1 eq) on rink amide MBHA resin were deprotected 2 x 10 min with 20 v/v% piperidine in DMF. **UPy-C_6_-U-C_12_-Ur-PEG_12_-C_2_-COOH** (22.8 mg, 0.02 mmol, 2 eq) was dissolved in 0.2 mL chloroform. 2,4,6-trimethylpyridine (10.6 µL, 0.08 mmol, 8 eq) and 1-[Bis(dimethylamino)methylene]-1H-1,2,3-triazolo[4,5-b]pyridinium 3-oxide hexafluorophosphate (HATU) (8.8 mg, 0.02 mmol, 2eq) were dissolved in 0.1 mL DMF, respectively, added to the **UPy-C_6_-U-C_12_-Ur-PEG_12_-C_2_-COOH** mixture and stirred for 30 min. After pre-activation the reaction mixture was added to the peptides on resin and reacted overnight under shaking conditions at room temperature. The UPy-peptides were cleaved from the resin using a 96.5:2.5:1 v/v% TFA:H_2_O:TIS mixture. The cleavage mixture was precipitated in ice-cold diethyl ether. The precipitate was collected, dissolved in water and lyophilized yielding 11.3 mg **UPy-PHSRN** (63%) as a fluffy white solid.


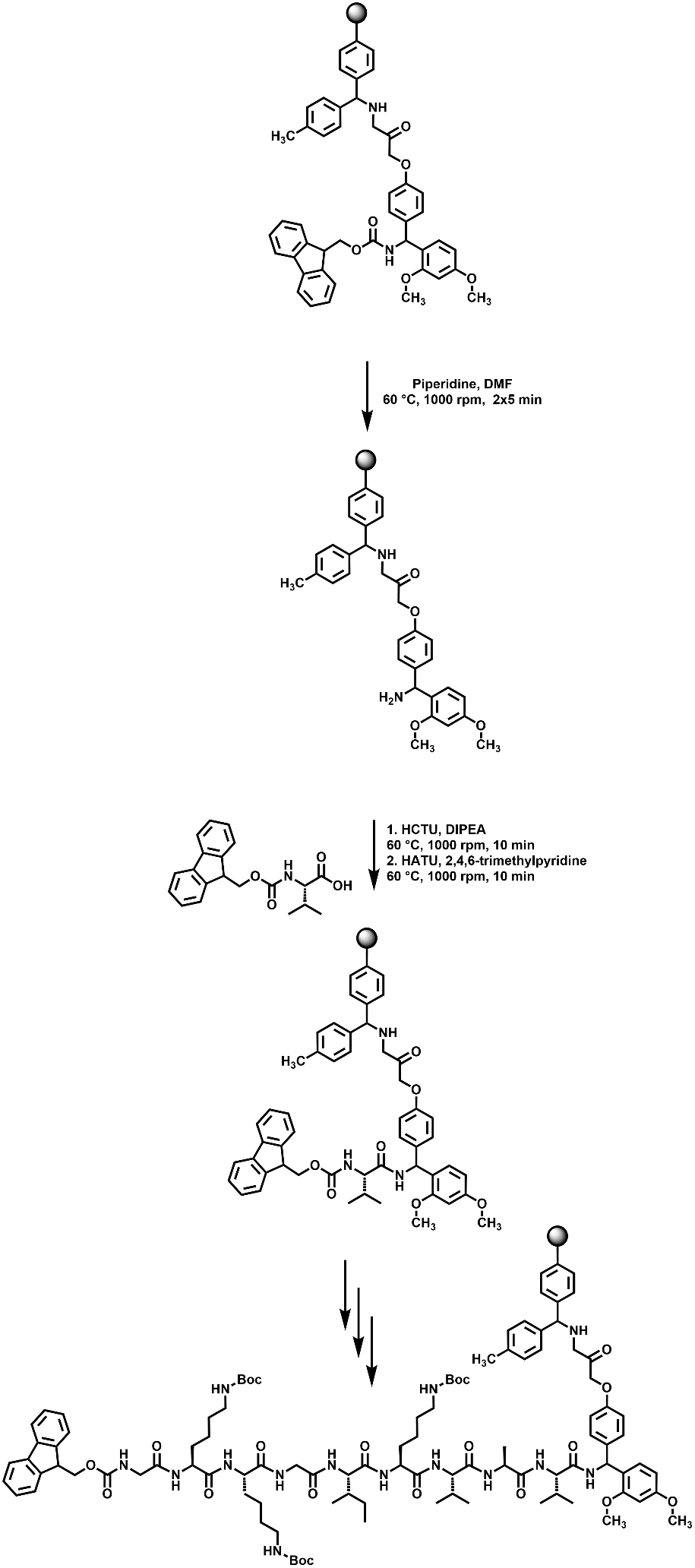


**Scheme S1:** Synthesis of **Fmoc-IKVAV.**


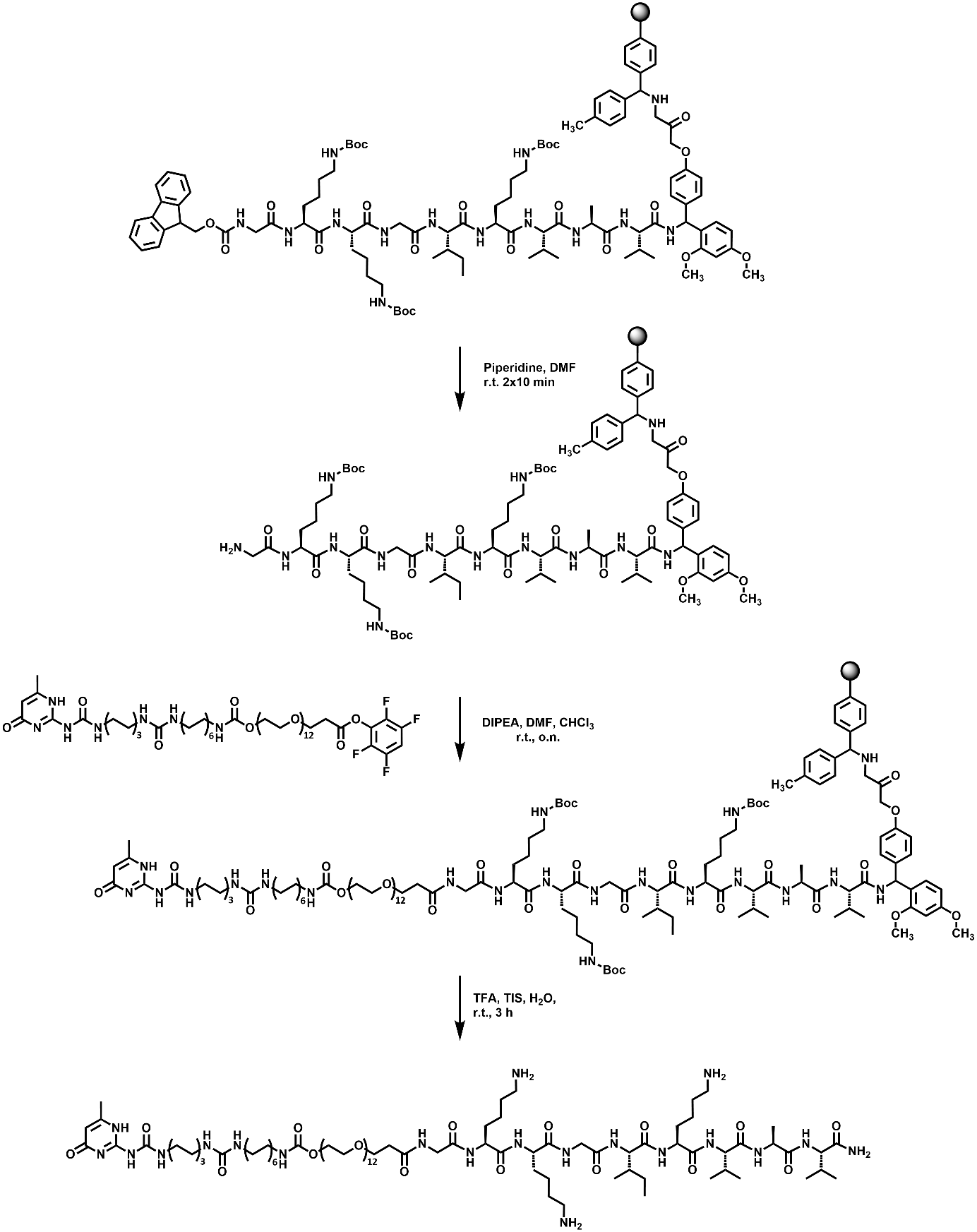


**Scheme S2:** Synthesis of **UPy-IKVAV.**


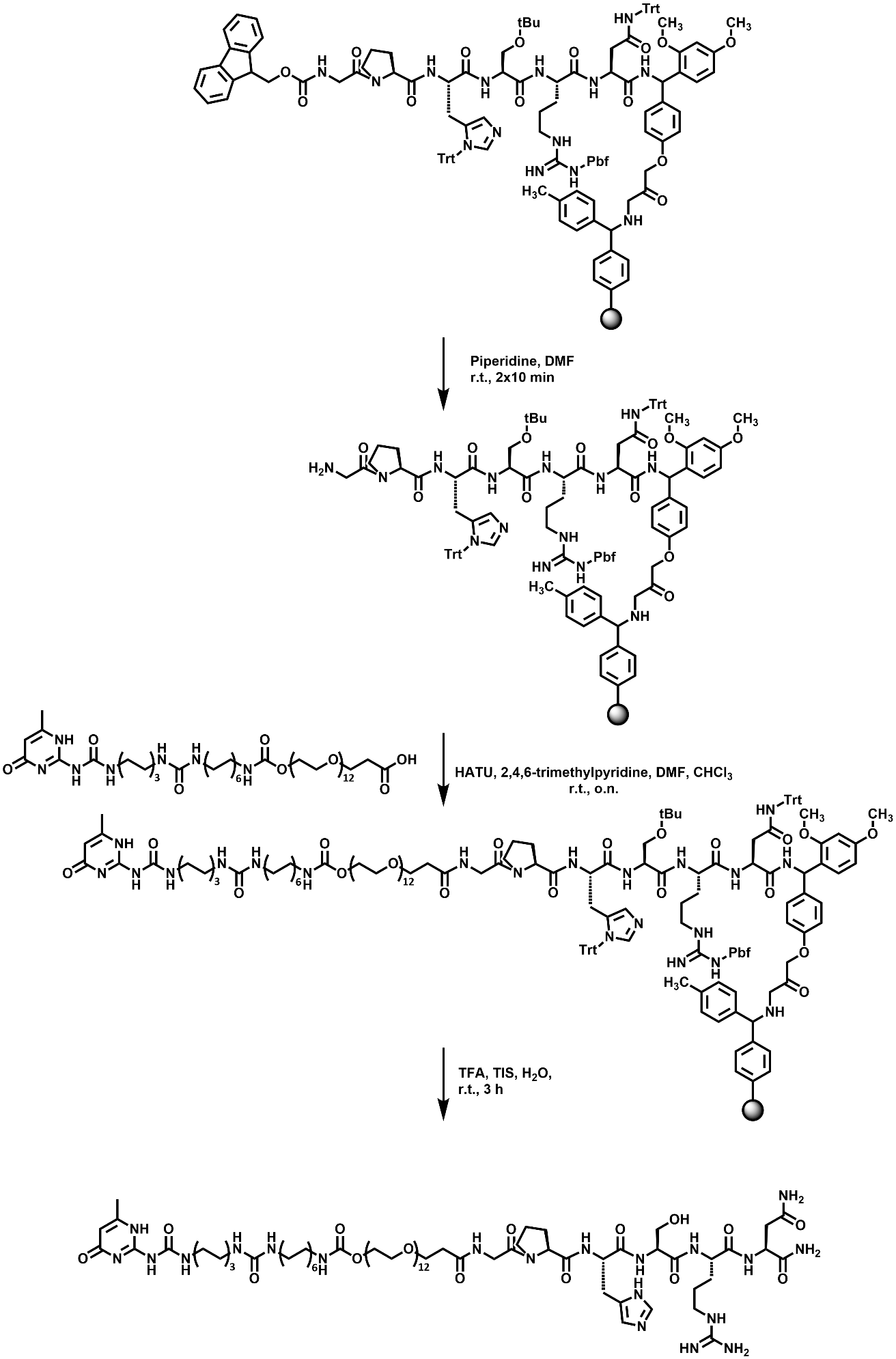


**Scheme S3**: Synthesis of **UPy-PHSRN.**


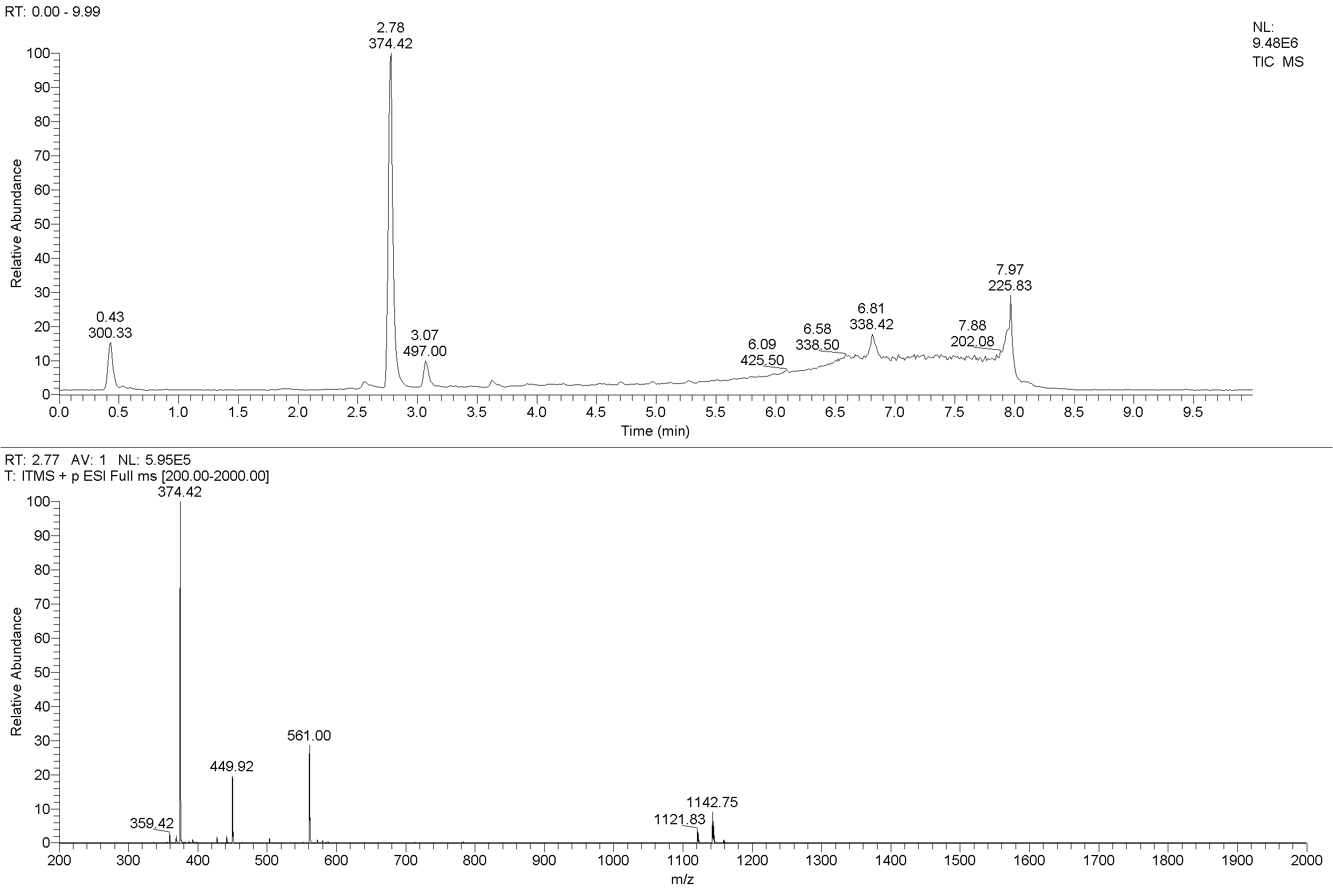


**Figure S1:** LC-MS analysis of **Fmoc-IKVAV** (C_56_H_89_N_13_O_10_): calc. MW = 1104.41 g mol^-1^, LC-MS(ESI) t_r_ = 2.78 min, m/z found: 1142.75 [M+K]^+^, 1121.83 [M+NH_4_]^+^, 561.00 [M+H+NH_4_]^2+^, 449.92 [M-Fmoc+H+NH_4_]^2+^, 374.42 [M+2H+NH_4_]^3+^.


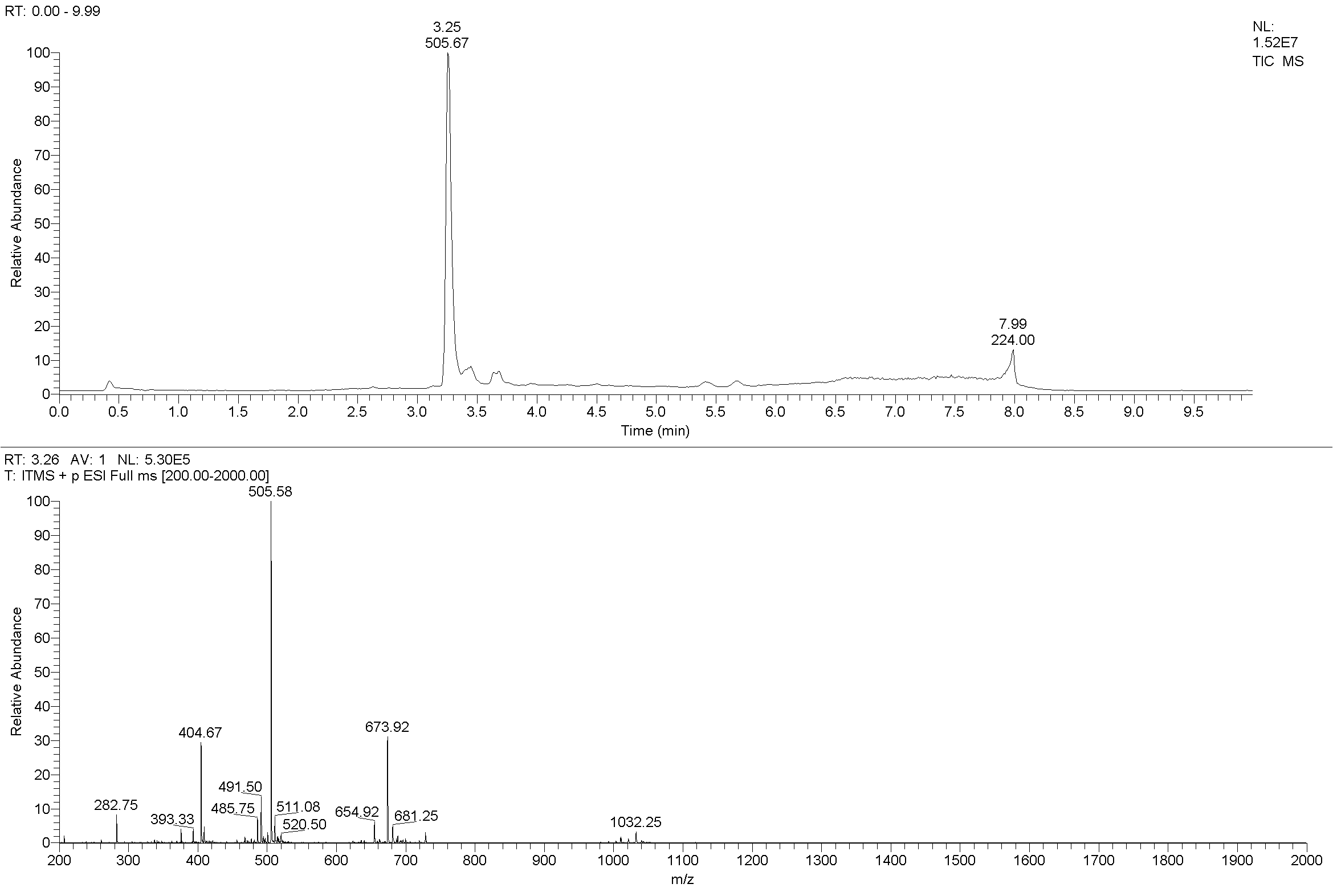


**Figure S2:** LC-MS analysis of **UPy-IKVAV** (C_94_H_176_N_20_O_27_): calc. MW = 2018.56 g mol^-1^, LC-MS(ESI) t_r_ = 3.25 min, m/z found: 1032.25 [M+2Na]^2+^, 673.92 [M+3H]^3+^, 505.58 [M+4H]^4+^, 404.67 [M+5H]^5+^.


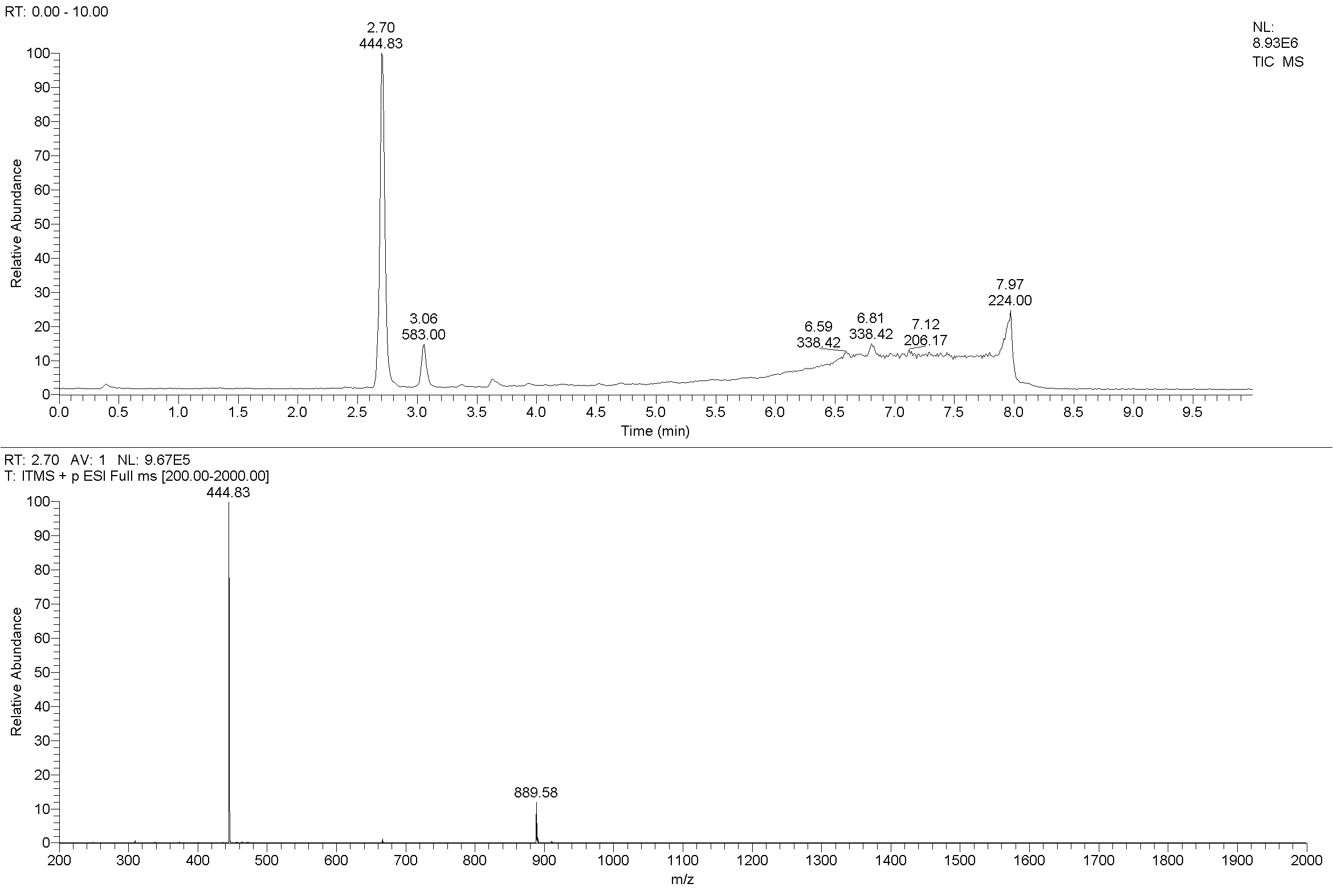


**Figure S3:** Liquid chromatography-mass spectrometry (LC-MS) analysis of **Fmoc-PHSRN** (C_41_H_53_N_13_O_9_): calc. MW = 871.96 g mol^-1^, LC-MS(ESI) t_r_ = 2.70, m/z found: 889.58 [M+NH_4_]^+^, 444.83 [M+H+NH_4_]^2+^.


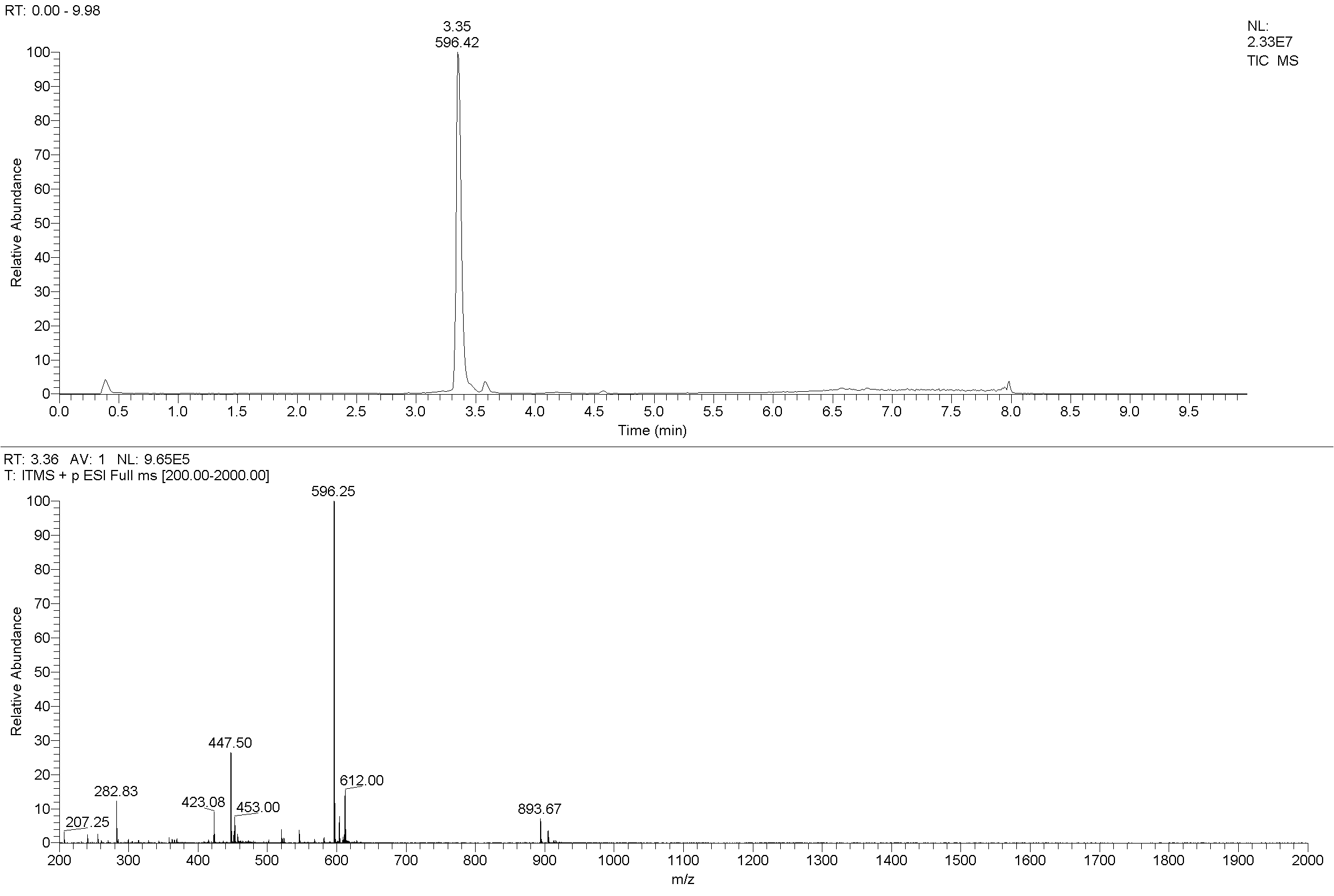


**Figure S4:** LC-MS analysis of **UPy-PHSRN** (C_79_H_140_N_20_O_26_): calc. MW = 1786.10 g mol^-1^, LC-MS(ESI) t_r_ = 3.35, m/z found: 893.67 [M+2H]^2+^, 596.25 [M+3H]^3+^, 447.50 [M+4H]^4+^.

1. **Supramolecular hydrogels: concentrations and molar ratios**

**Table S1:** Table showing the different samples that are used with molecular ratios between the supramolecular monomers for 1.2 w/v% hydrogels with M/B=80/1 that contain different types of bioactive UPy (1 mM).

|  | **Concentration [mg/mL]** | **Molar concentration [mM]** | **Molar ratio [-]** |
| --- | --- | --- | --- |
| **Sample ID** | **M - B - bioactive** | **M - B - bioactive** | **M - B - bioactive** |
| **UPy-cRGD** | 9.66– 1.27 – 1.72 | 8.1 – 0.115 – 1 | 70 – 1 – 9 |
| **UPy-GFOGER** | 9.66 – 1.27 – 4.47 | 8.1 – 0.115 – 1 | 70 – 1 – 9 |
| **UPy-IKVAV** | 9.66 – 1.27 – 2.02 | 8.1 – 0.115 – 1 | 70 – 1 – 9 |

**Table S2:** Table showing the different samples that are used with molecular ratios between the supramolecular monomers for samples that dual ligands, 1 mM **UPy-cRGD** with 1 mM of additional bioactive UPy (**UPy-IKVAV**, **UPy-PHSRN**) in the 1.2 w/v% hydrogels with M/B=80/1.

|  | **Concentration [mg/mL]** | **Molar concentration [mM]** | **Molar ratio [-]** |
| --- | --- | --- | --- |
| **Sample ID** | **M - B – cRGD - bioactive** | **M - B – cRGD – bioactive** | **M - B – cRGD - bioactive** |
| **UPy-IKVAV** | 8.5 – 1.27 – 1.72 – 2.02 | 7.1 – 0.115 – 1 – 1 | 61 – 1 – 9 - 9 |
| **UPy-PHSRN** | 8.47 – 1.27 – 1.72 – 1.79 | 7.1 – 0.115 – 1 – 1 | 61– 1 – 9 – 9 |

**Table S3:** Table showing the different samples that are used with molecular ratios between the supramolecular monomers upon decreasing **UPy-IKVAV** concentration, in the 1.2 w/v% hydrogels with M/B=80/1.

|  | **Concentration [mg/mL]** | **Molar concentration [mM]** | **Molar ratio [-]** |
| --- | --- | --- | --- |
| **Sample ID** | **M - B - IKVAV** | **M - B – IKVAV** | **M - B – IKVAV** |
| 0.01 mM | 10.9 – 1.27 – 0.02 | 9.1 – 0.115 – 0.01 | 79 – 1 – 0.1 |
| 0.1 mM | 10.7 – 1.27 – 0.2 | 9 – 0.115 – 0.1 | 78 – 1 – 1 |
| 1 mM | 9.66 – 1.27 – 2.02 | 8.1 – 0.115 – 1 | 70 – 1 – 9 |

**Table S4:** Table showing the different samples that are used with molecular ratios between the supramolecular monomers for samples that contain 1 mM **UPy-IKVAV** with M/B=80/1, upon changing the total UPy hydrogel concentration.

|  | **Concentration [mg/mL]** | **Molar concentration [mM]** | **Molar ratio [-]** |
| --- | --- | --- | --- |
| **Sample ID** | **M - B - IKVAV** | **M - B - IKVAV** | **M - B - IKVAV** |
| 0.6 w/v% | 4.18 – 0.64 – 2.02 | 3.5 – 0.06 – 1 | 63 – 1 – 17 |
| 1.2 w/v% | 9.66 – 1.27 – 2.02 | 8.1 – 0.115 – 1 | 70 – 1 – 9 |
| 2.5 w/v% | 20.4 – 2.54 – 2.02 | 17.1 – 0.23 – 1 | 75 – 1 – 4 |

**Table S5:** Table showing the different samples that are used with molecular ratios between the supramolecular monomers upon changing hydrogel dynamics by varying the M/B UPy ratio from the normal (M/B=80/1) towards the more dynamic (M/B=9/1) condition, while keeping a fixed total UPy concentration of 1.2 w/v% with 1 mM **UPy-IKVAV**.

|  | **Concentration [mg/mL]** | **Molar concentration [mM]** | **Molar ratio [-]** |
| --- | --- | --- | --- |
| **Sample ID** | **M - B - IKVAV** | **M - B - IKVAV** | **M - B - IKVAV** |
| Normal **M/B=80/1** | 9.66 – 1.27 – 2.02 | 8.1 – 0.115 – 1 | 70 – 1 – 9 |
| Dynamic **M/B=9/1** | 4.18 – 5.52 – 2.02 | 3.5 – 0.5 – 1 | 7 – 1 – 2 |

1. **Supplementary data**


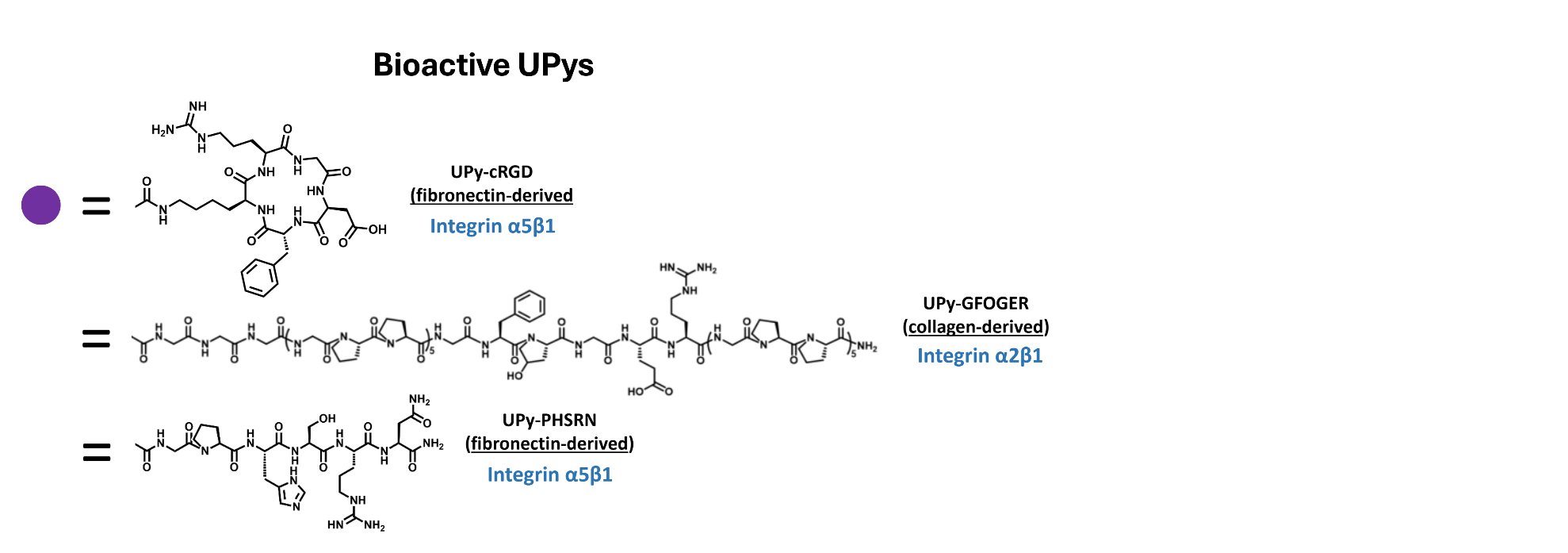


**Figure S5**: **Chemical structures of other bioactive UPys: UPy-cRGD, UPy-GFOGER and UPy-PHSRN.**


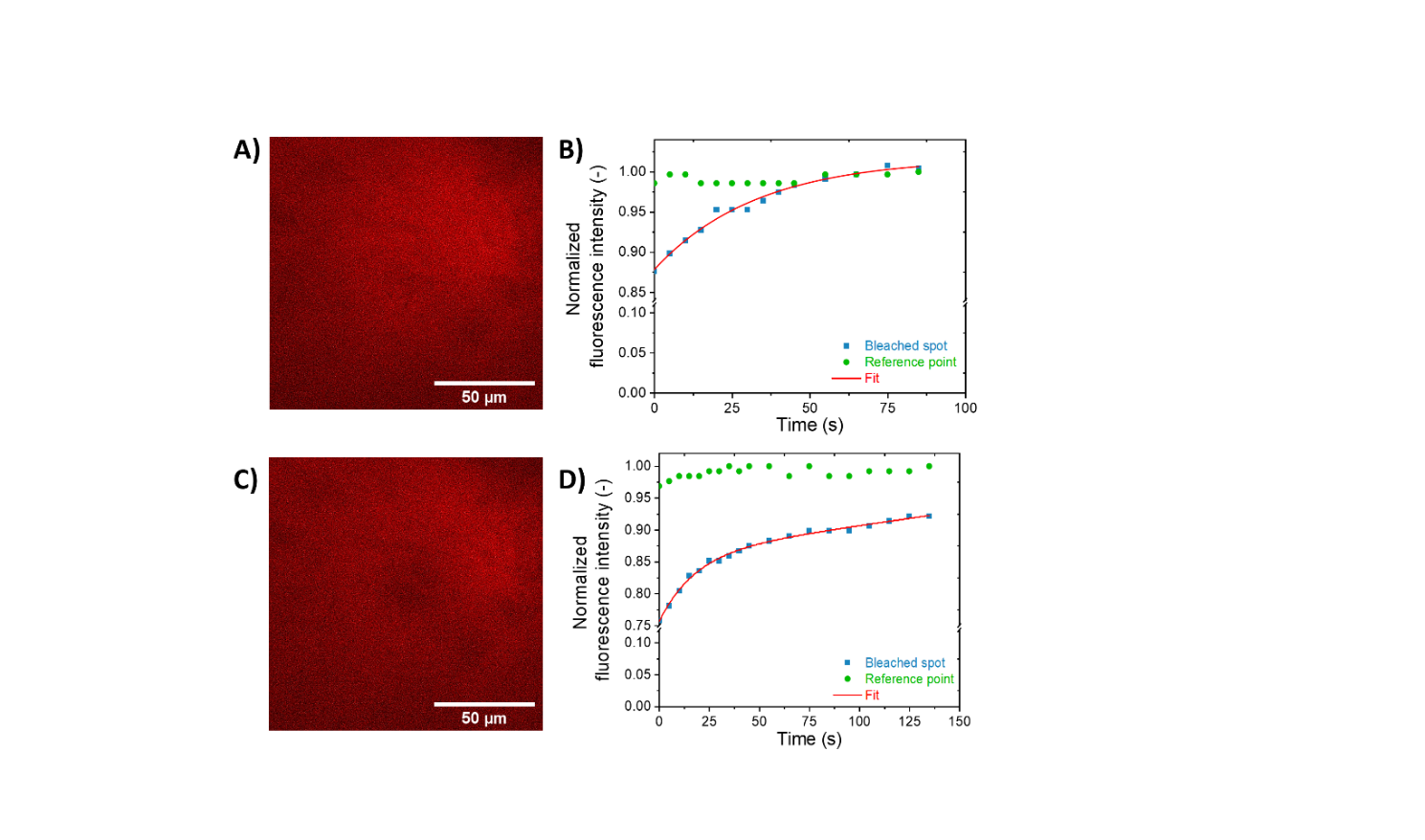


**Figure S6**: **Diffusion experiments of TS2/16 antibody through UPy hydrogels using fluorescence recovery after photobleaching (FRAP) experiment.** **A,C)** Fluorescent confocal microscopy images directly before photobleaching showing a homogeneous distribution of the Alexa647-labeled antibody through the gel for both the **A)** 1.2 and **C)** 2.5 w/v% gel. Scale bar = 50 $\mu$m. **B,D)** Normalized fluorescence recovery over time for the **B)** 1.2 and **D) 2**.5 w/v% gel. Fluorescence recovery in the bleached spot is fitted by a single exponential growth model. All samples contain 0.5 mg/mL of TS2/16-Alexa647 antibody.


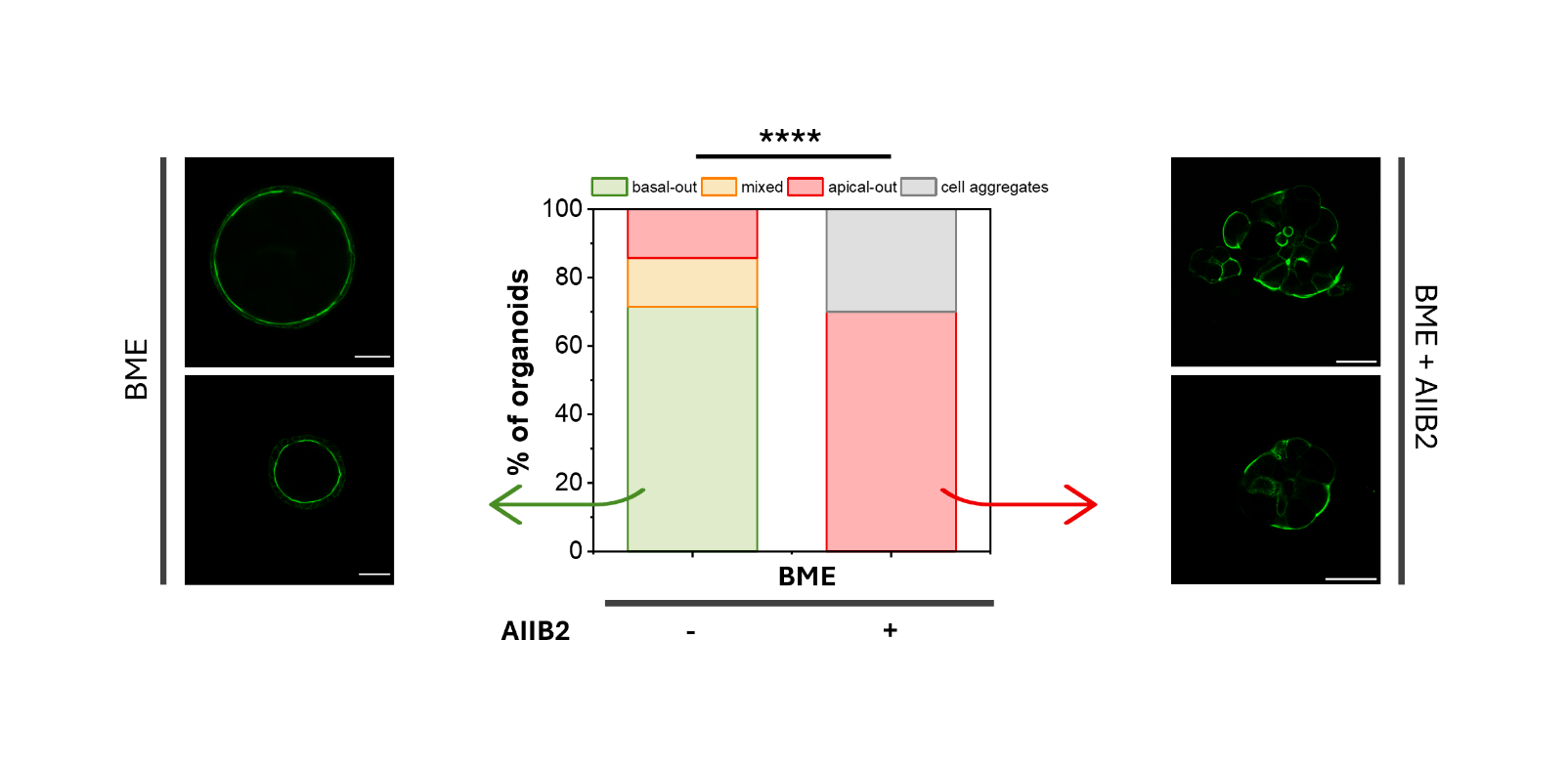


**Figure S7:** **The culture of intestinal organoids in Basement Membrane Extract (BME), a Matrigel equivalent, and in presence of integrin β1 blocking antibody.** Quantification of the frequencies of the differently polarized organoids that were formed inside BME and in presence of AIIB2 integrin β1 blocking antibody after 5 days of culture, including representative fluorescence confocal images of the different conditions. In green organoids with correct, basal-out polarity, in orange mixed polarity, in red reversed, apical-out polarity and in grey cellular aggregates. Green is F-actin, an apical marker. Scale bar = 50 µm. The categorical organoid polarity data was subjected to a 𝜒2 test by comparing the groups individually to each other. n=13-30 organoids per condition. Differences were considered as statistically significant when p < 0.05, with * p < 0.05; ** p < 0.01; *** p <0.001; **** p < 0.0001.


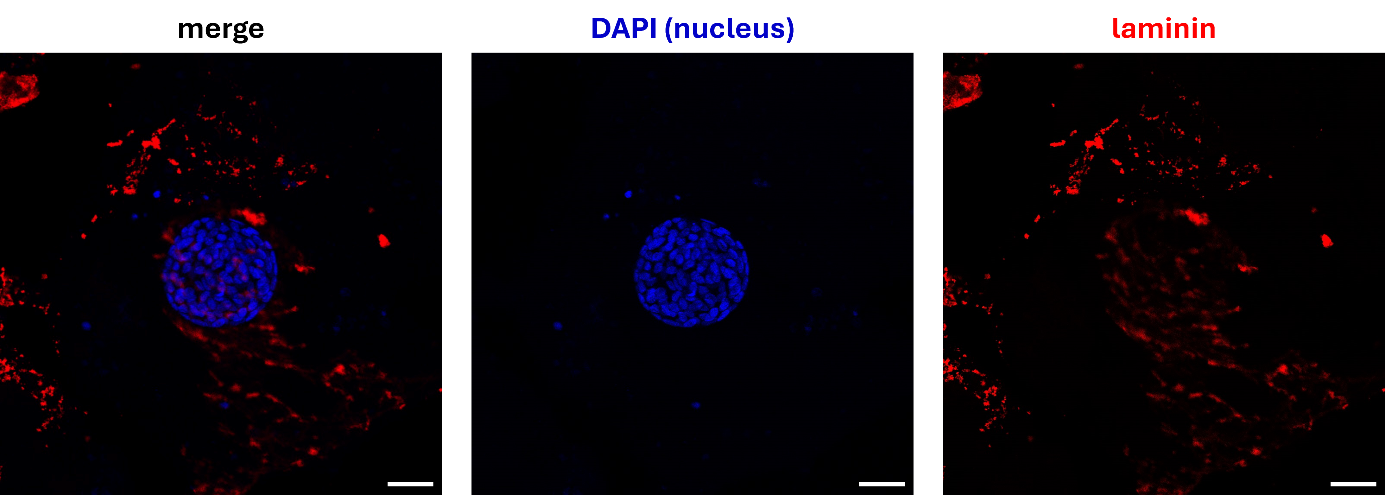


**Figure S8:** **The culture of intestinal organoids in BME, a Matrigel equivalent, and staining of extracellular laminin.** Intestinal organoids were cultured for 5 days inside BME. Blue is DAPI (nucleus) and in red extracellular laminin. Scale bar = 50 µm.


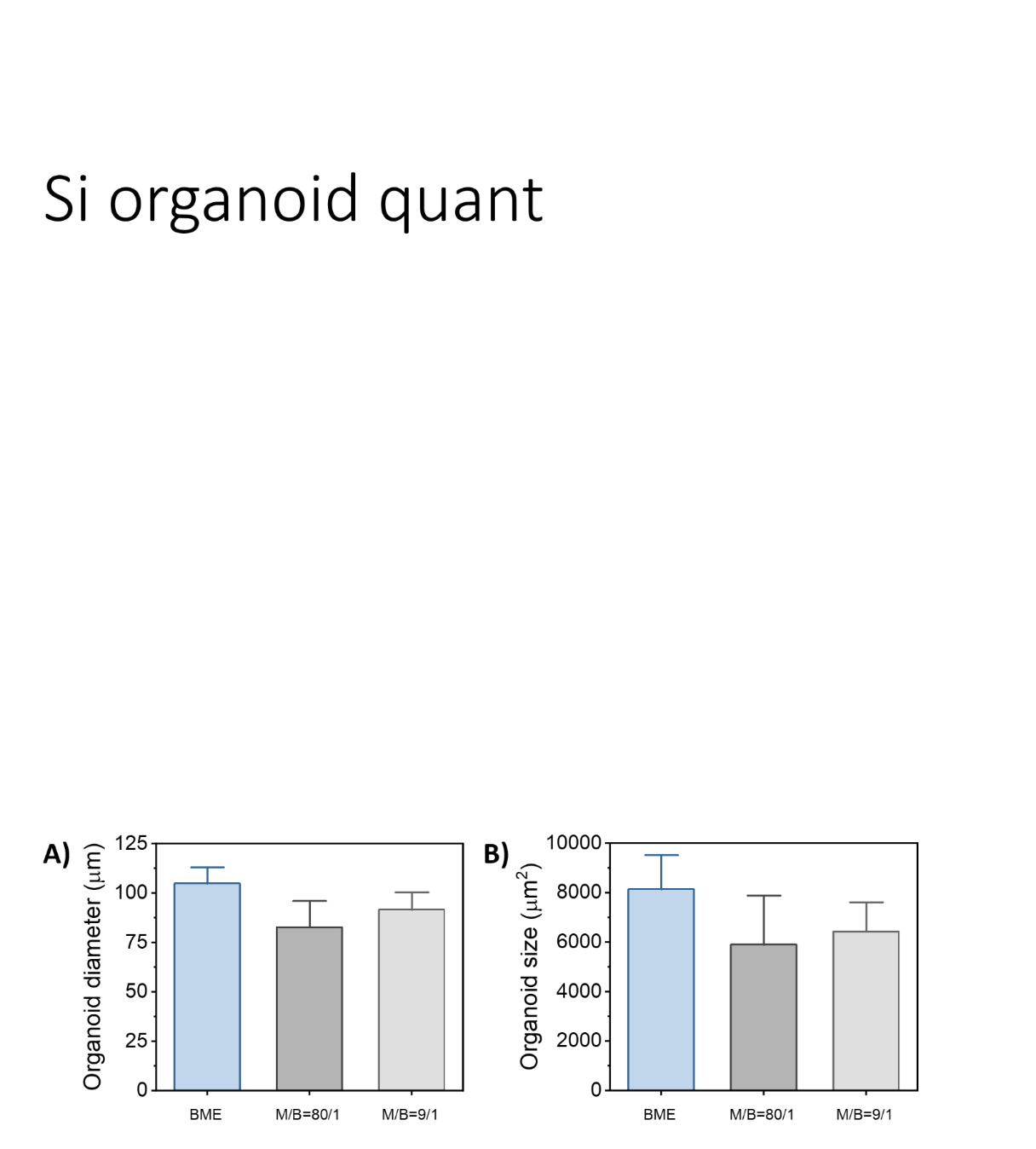


**Figure S9:** **Quantifications on the influence of gel dynamics on intestinal organoids polarity.** **A)** Quantification of organoid diameter in µm and **B)** organoid size in µm^2^, for intestinal organoids cultured in normal (M/B=80/1) and dynamic (M/B=9/1) UPy gels as well as in BME. n=13-30 organoids per condition. Quantified data is plotted with mean and standard error of the mean (SEM).
